# Advances of nano drug delivery system for the theranostics of ischemic stroke

**DOI:** 10.1186/s12951-022-01450-5

**Published:** 2022-05-31

**Authors:** Wei Lv, Yijiao Liu, Shengnan Li, Lingyan Lv, Hongdan Lu, Hongliang Xin

**Affiliations:** 1 Department of Pharmacy, The Jiangyin Clinical College of Xuzhou Medical University, 214400 Jiangyin, China; 2 Department of Pharmaceutics, School of Pharmacy, Nanjing Medical University, 211166 Nanjing, China

**Keywords:** Ischemic stroke, Brain targeting, Nanoparticle-based drug delivery, Blood brain barrier

## Abstract

From the global perspective, stroke refers to a highly common cause of disability and death. Ischemic stroke (IS), attributed to blood vessel blockage, preventing the flow of blood to brain, acts as the most common form of stroke. Thus far, thrombolytic therapy is the only clinical treatment for IS with the approval from the FDA. Moreover, the physiology barrier complicates therapeutically and diagnostically related intervention development of IS. Accordingly, developing efficient and powerful curative approaches for IS diagnosis and treatment is urgently required. The advent of nanotechnology has brought dawn and hope to better curative and imaging forms for the management of IS. This work reviews the recent advances and challenges correlated with the nano drug delivery system for IS therapy and diagnosis. The overview of the current knowledge of the important molecular pathological mechanisms in cerebral ischemia and how the drugs cross the blood brain barrier will also be briefly summarized.

**Graphical Abstract:**

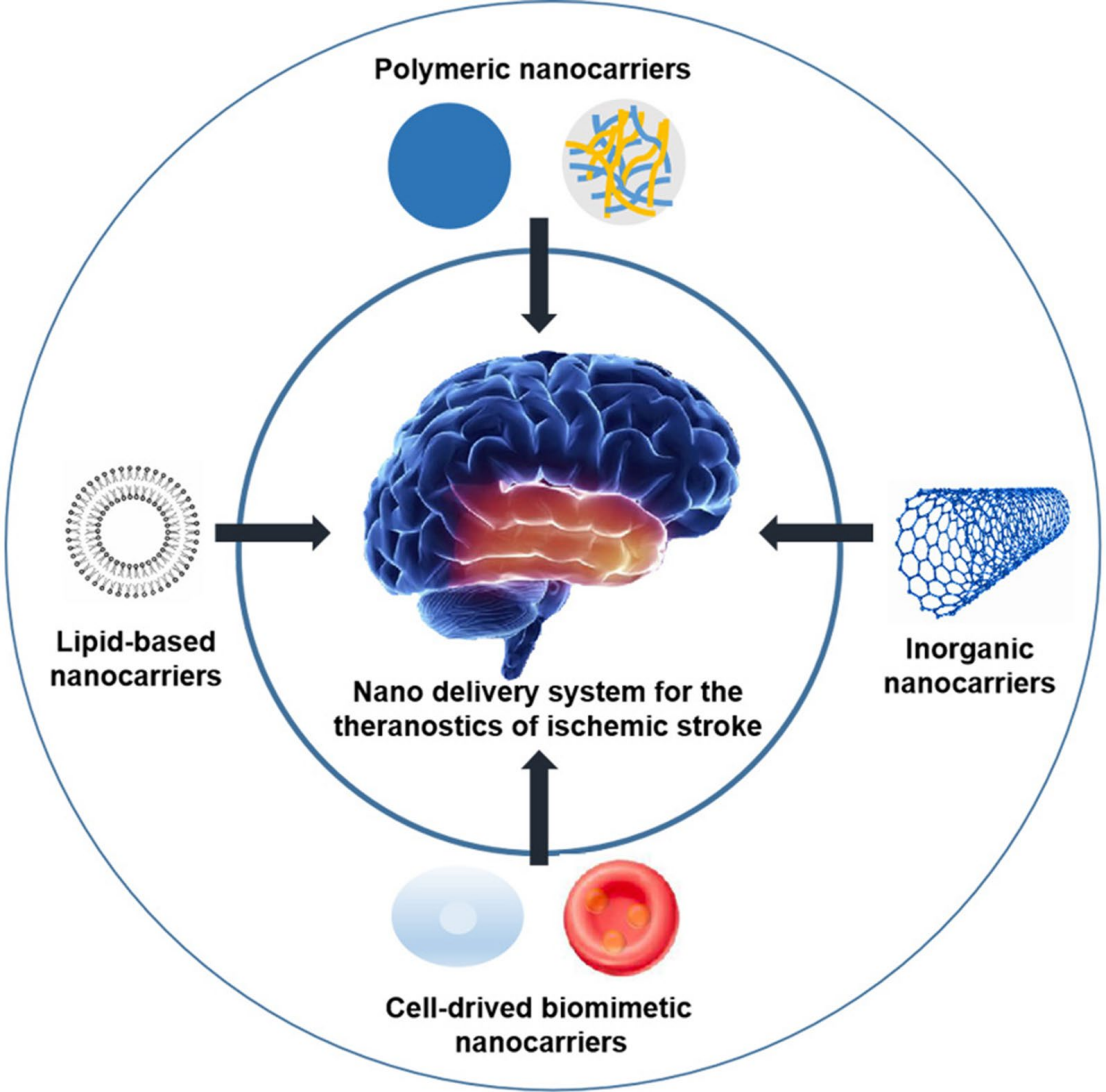

## Introduction

Stroke is a brain disorder that is attributed to a dramatic blood vessel rupturing or blocking within the brain, thereby preventing the flow of blood to the brain. As impacted by a significant effect exerted by disease worldwide, stroke continues to be a main health issue in the globe, representing the 5th top reason for death within the USA as well as the third primary reason for potential life lost worldwide. Nearly a population of 15 million encounter stroke each year in the globe, with 33% caused to be permanently disable, and 40% caused to die [1, 2]. It is estimated that the number of stroke cases and related deaths will rise to 23 million and 7.8 million by 2030, respectively. Given to lately conducted meeting regarding the American Heart Association (AHA), nearly 87% of stroke is ischemic stroke (IS) [3]. Thus far, the diagnosing and treating processes for brain injuries have continued to be highly challenging, in particular few effective measures for IS therapy are available after decades of research in general [4, 5]. The optimal clinical treatment for IS is thrombolysis within the optimal treatment window of 4.5 h after the disease onset, as an attempt to restore cerebral blood flow perfusion [6]. The sole treatment of IS approved in the USA acts to clear the thrombus that blocks blood flow, either by using mechanical approaches or by employing intravenous tissue-type plasminogen activator (tPA). However, most patients of IS could not be well treated in time as impacted by the optimal short treatment window and risk of reperfusion injury, intracerebral bleeding and the rest negative influences [7, 8]. For hospital engaged within the GWTG-Stroke scheme, merely 24.7% of patients with IS could present themselves within 3 h, let alone eligible for tPA [9]. Thus, IS pathology and etiology are required to be taken in account at the identical time for discovering appropriate curative drugs and exploring possible curative molecule targets in terms of the development of neuroprotective drugs.

To date, though several pre-clinical therapies have reported that neuroprotectants (including apoptosis suppressors and antioxidant) exert good effects for treating IS, none of them are efficacious in humans [10]. The most significant difficulty refers to the delivery of curative compounds to the brain of the target, according to the fault of the majority of advanced neuroprotective agents for accessing the brain under a great amount [11]. The primary limit of drug delivery is the transferring process across the blood-brain barrier (BBB), whose presence can prevent the majority of neuroprotectants from the brain [12]. Consequently, new approaches exhibiting improved delivery efficiency are urgently required.

The central nervous system (CNS) has developed several barriers for protecting its invasion in circulating blood cell, neurotoxic molecule and pathogen. Among the mentioned, BBB is the most extensive and exclusive, under the formation of brain microvasular endothelial cells (BMECs) lining the micro-scale brain vessels, followed by tightly correlated astrocyte and pericyte. It was previously shown that BBB is a dynamic and functional neurovascular unit well separating CNS from circulating mechanism and protecting CNS against possibly adverse toxin, chemical and infection within blood [13, 14]. In addition, IS could initiate the opening of BBB in a short period (minutes to hours), accompanied with a second reopening under a prolonged period (hours to days) [15, 16]. The reperfusion of the ischemic areas is essential for mitigating brain injuries, whereas such an event is cable of causing ischemia-reperfusion injury as well, thereby triggering subsequent BBB opening again. The dysfunction of BBB shows the primary correlation to tight junction protein dysregulation including claudins and membrane-correlated guanylate kinase-like protein, occludin, and junctional adhesion molecule [17, 18]. The mentioned key junction proteins of BBB may be altered by oxidative stress resulting from the production of reactive oxygen species (ROS) after brain injury [19]. In particular, the BBB opening may be compromised by effective efflux pumps in the neuro-endothelium that return small molecules to circulation [20]. The loss of functional BBB results in serious complications, indicating that treatments for IS will have to contend with an intact BBB and should prevent its degradation [21]. A more effective insight in brain injury pathology and BBB permeability should be gained to develop optimized curative interventions. On the whole, the BBB leakage and changes in BBB receptor expression are likely to endow extensive chances of curative nanocarriers, as an attempt to exploit the brain penumbra region to achieve probable neuro-protection and neurovascular unit maintenance.

As a primary disease for mortality and morbidity globally, IS has aroused surprisingly less attention of the nanobiotechnology research compared with tumor or diabetes [22, 23]. The potential benefits of nanobiotechnologies (including extending the blood half-life of molecules by creating a protective outer layer [24] and designing both for accumulation in brain tissues and the intact BBB transition [25]) of brain injury uses are sufficient and are likely to present new clinically related option for addressing existing limits. The present review is inspired for briefly describing the molecular pathological mechanisms and the limits of existing treatments of IS. More importantly, this review summarizes the latest research on the systemic administration of nanocarriers for IS management. We would outline transport in the brain following IS and describe the effect of the general physical properties of nanocarrier and some approaches for active targeting. Lastly, we would provide an overview of the future directions in the field by suggesting a number of design parameters for nanomedicine for IS management, which may offer guidance and useful information to clinically translate nano-interventions.

## Pathophysiology of IS

**Fig. 1:**
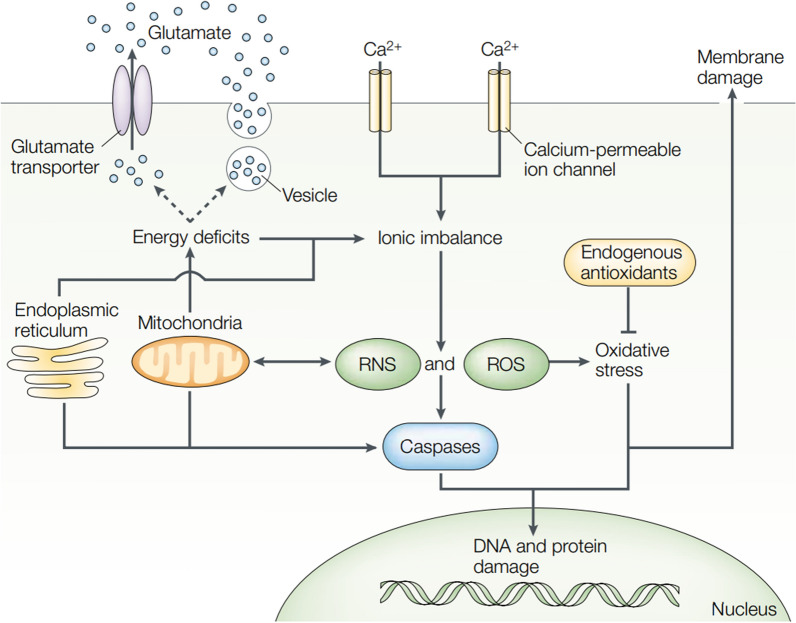
Main channels correlated with ischemic cell apoptosis: apoptotic-like system, oxidative and nitrosative stresses, ionic imbalance and excitotoxicity. Reproduced with permission from [29]. Copyright 2003, Springer Nature

IS, attributed to the flow of blood blocked to the brain, is the most common type of stroke [26]. Brain injury resulting from IS refers to a complex cascade of pathological biochemical reactions. First, insufficient oxygen and energy availability cause several stress responses in the acute phase, which lead to the up-regulation of cytokines and ROS and the activation of microglia and astrocytes. Subsequently, the cytokines secreted by glial cells destroy BBB integrity and the white blood cells covering neutrophils would achieve the migration to brain parenchyma, aggravating inflammation-related response, thereby causing death of neuron, BBB injury and brain edema. In the chronic recovering period, the macrophage enters the cerebral ischemia region for participating in neuronal regenerating process [27, 28]. It is generally considered that the vital effect exerted by inflammation, apoptotic-like channels, oxidative stress and glutamate excitotoxicity are responsible for the main progress of ischemic brain injuries [29, 30] (Fig. 1).

### Oxidative stress

Oxidative stress refers to part of downstream consequences of excitotoxicity induced by the production of ROS and is generally indicated to activate the channels of apoptosis, necrosis and autophagy, thereby facilitating the final infarct size in brain [31]. The intracellular calcium overload, in particular in the mitochondria, will stimulate calmodulin-dependent enzyme (e.g., nitric oxide (NO) synthase) and Ca^2+^-dependent enzyme excessively (e.g., protease, phospholipase and endonuclease) [32]. In cerebral ischemia, superoxide, production of ROS exhibiting great efficiency, is at such high levels that the metabolism ability exhibited by superoxide dismutase is submerged. Such mechanism is likely to be driven by inadequate oxygen supply, in particular after reperfusion. Lastly, the mass reproduction of ROS can damage cellular macromolecules directly or by serving as intermediates in diverse signal transduction channels, consisting of protein oxidation, lipid peroxidation and DNA fragmentation, thereby probably triggering the further brain tissue injury [33, 34].

### Glutamate-mediated excitotoxicity

It is generally acquired that the first change after cerebral ischemia is a down-regulation within cerebral blood flow, thereby causing inadequate delivery of oxygen and glucose. As impacted by oxidative phosphorylation acts as the major energy production source, the decreased glucose and oxygen supplies will decouple oxidative phosphorylation, followed by the lessened ATP production as well as ion pump function injury [30]. The decreased levels of intracellular ATP could hinder ion gradient fault through ATP-dependent ion channel termination including the ATP-dependent Na^+^/K^+^-ATPase, thereby depolarizing the neuronal membranes with Na^+^ abnormal influx and K^+^ efflux [35]. The insufficient ATP will also lead to the degradation of protein and lipid in the cell matrix, destroying the integrity exhibited by the cells. In addition, voltage-gated calcium channels are activated by depolarization, reversing Na^+^/Ca^2+^ exchanger orientation and introducing Ca^2+^ to cells, which causes excitotoxic amino acids release, including glutamate. The uncontrolled accumulation of glutamate can overstimulate the metabotropic glutamate receptor and broadly stimulate the receptors of N-methyl-D-aspartic acid (NMDA) and α-amino-3-hydroxy-5-methyl-4-isoxazole-propionicacid (AMPA), thereby disrupting calcium homeostasis of CNS [36]. Then the energy reserve will soon run out for sequestering the rise of Ca^2+^ content in cells [37]. Calcium overload will cause a deleterious cascade of metabolic events through systems under the mediation of oxidative stress including inflammation and apoptosis, causing neuronal cell apoptosis as well as infarction development [38].

### Apoptotic-like channels

Membrane integrity loss and organelle failure refer to the critical systems of cell apoptosis within ischemia. Yet existing studies have obviously implicated systems following apoptotic-like channels and cascade, in particular in the penumbra. Both caspase-dependent and caspase-independent systems are presented. The cell apoptosis channels show correlations with an apoptotic-like system within cerebral ischemia. For instance, Cytochrome c released from mitochondria receives the modulation to be anti-apoptotic Bcl2 family member. Cytochrome c release can activate downstream caspases through apoptosis body formation and caspase stimulation is modulated indirectly by caspase secondary catalyst obtained from mitochondria through hindering inhibitors of apoptotic protein (IAP). Effector caspases (Caspase 3 and 7) target substrate, dismantling cells through the process to cleave metabolic, repair, cytoskeletal, homeostatic and cell signaling proteins. Caspase-independent cell apoptosis is likely to be vital. According to a system, poly-(ADP ribose) polymerase (PARP) stimulation boosts the release of apoptosis-inducing factor (AIF), thereby being translocated to the nucleus, while the system of which binding to DNA and boosts cell apoptosis based on a system awaiting elucidation.

### Inflammation

Inflammation starts few hours when cerebral ischemia occurs. The IS can trigger the release of a wide range of inflammation-related markers including inducible nitric oxide synthase (iNOS) and eicosanoids, cell adhesion molecules (immunoglobulins, selectins and integrins), cytokines (TGF-β, IL-6, the interleukins IL-1 and TNF-α) [39]. Leukocyte and microglial cells will adhere to the brain endothelial cells and release matrix metalloproteinase and nitric oxide, which further increase the death of neurons [40, 41].

## Traditional treatments for IS

At present, the routine therapies to manage IS and correlated complications largely concentrated on drugs to remove the thrombus, as well as to minimize deleterious ischemic injuries to neurons *via* intravascular therapy or intravenous thrombolysis. The vital element determining the traditional therapy’s efficacy is rapid thrombolysis in a small window of time (3–4 h) for ensuring minimal brain impairment. Surgical intervention including mechanical endovascular thrombectomy has also been developed to restore blood flow in the occlusion of an intracranial vessel. Nevertheless, the mentioned intervention has not had the ability to prevent death or disability in primary cases. Table 1 lists the promising conventional drugs that are useful in stroke therapy and their mechanisms of action or characteristics.

### Thrombolytic and antiplatelet agents

Until recently, intravenous administration of rt-PA in terms of thrombolysis continues to be the only commercially-available treatment approved by the FDA. This therapy is largely determined by the narrow time for being effective (less than 30% of stroke patients) and also increases the risk of intracerebral hemorrhages [42]. Recently, some newly developed thrombolytic agents have been well developed, including the fibrin-particular tissue plasminogen activator (i.e., tenecteplase, lanoteplase, desmoteplase, reteplase, staphylokinase, etc.) as well as chimeric plasminogen activator. But almost all the mentioned thrombolytic agents exhibit numerous defects restricting the applications for treating IS [43, 44].

Secondary prevention of stroke can also be done by antiplatelet therapy, anticoagulants accompanied with or without surgical treatment. The provision of anticoagulants like aspirin and statins for the patients refers to the sole cheap option, whereas the mentioned antiplatelet drugs are almost adopted to be precaution for subsequent or probable episodes, and capable of repairing or shielding the influenced region from more injury in no circumstances [45]. Therefore, there is an increased need for directional delivery of these agents without counteracting the properties exhibited by rapid release and clot dissolution.

### Neuroprotective agents

As a promising curative approach, the neuroprotective agents aim at improving survival of neurons after ischemic stroke to increase the curative window, as well as inducing neurological repair to improve functional outcomes. For this reason, they are commonly used in combination with drugs of other categories. Some classes of neuroprotectants consist of free radical scavengers, γ-aminobutyric acid (GABA) agonists [46], calcium channel blockers [47], glutamate antagonists and nitric oxide antagonists [48, 49]. Over the last twenty years, more than 1000 neuroprotective agents have been developed, 10% of which have been progressed to clinical trials [50]. But unfortunately, though many of the mentioned agents have been proven to exhibit high effectiveness in animal models of IS, none of them have revealed convincing benefits in clinical trials [51]. The low permeability of neuroprotectants across BBB and poor simulation of animal model comparing to the patients may contribute to the failures of clinical transformation [52, 53]. So approaches to increase neuroprotectants accumulation in ischemia site will not only greatly ameliorate the curative efficiency, but also contribute to their clinical translation.

**Table 1:** List of traditional drugs for IS

Types	Representative drugs	Mechanism or characteristics
Thrombolytic agents	Tissue plasminogen activator (t-PA)	Employed to dissolve coronary and cerebral vascular thrombus. Binds to the clot surface and activates plasminogen bound to fibrin.
	Tenecteplase (TNK-t-PA)	Derived from t-PA after mutations at the three regions T103, N117 and KHRR296–299.
	Lanoteplase	Derived from t-PA after deletion of fibronectin finger-like and epidermal growth factor domains.
	Desmoteplase	Derived from the saliva of vampire bats and structurally similar to alteplase.
Antiplatelet agents	Aspirin	COX-1 inhibitor
	Clopidogrel and Ticagrelor	Selective and irreversible inhibition of binding of adenosine diphosphate (ADP) to platelet receptors (P2Y12).
	Tirofiban, Eptifibatide and Abciximab	Platelet glycoprotein IIb / IIIa receptor antagonist.
	Treprostinil and Iloprost	Prostacyclin inhibitor.
Neuroprotective agents	Memantine	Inhibit excessive glutamate and reduce cerebral infarction, without affecting the physiological activation of NMDA receptor.
	ZL006	Block the ischemia induced nNOS-PSD-95 association selectively.
	Apocynin	An antioxidant and inhibitor of NOX2 and NADPH by reducing the production of ROS.
	NXY-059	A scavenger of free radicals, but with low permeability to BBB.
	Edaravone	A free radical scavenger and inhibits lipid peroxidation-mediated neuronal damage.
	Ethylenedimainetetraacetic acid (EDTA)	Chelate calcium from atherosclerotic plaques and serve as antioxidants.
	Nimodipine, Nifedipine and Amlodipine	Inhibit atherosclerotic plaque deposits and exhibit anti-hypertensive action as well as neuroprotective agents.
	Erythropoietin	Inhibit apoptosis and the inflammatory cascades.
	Diazepam and Chlormethiazole	GABA receptor agonists and attenuate stroke-associated neuroinflammation.

## The Challenges in traditional treatments

It is obvious from clinical trials or reports that a single drug is ineffective, but a mixture of multiple therapeutic drugs with different targets are required for better prevention and management of IS. The major toxicity of systemic administration of conventional drugs (i.e., thrombolytic agent) is the activation of plasminogen in regions with normal circulation leading to hemorrhagic complications [54]. Besides targeting drugs for specific delivery and co-delivery to ischemic areas, the temporal distribution of these agents is also a key factor. Therefore, site-specific delivery of the corresponding drugs is preferred for more effective management of ischemia stroke.

Another major challenge of stroke treatment is to bypass the physiological barriers (i.e., BBB and blood cerebrospinal fluid barrier (CBB)) raised by the brain. Moreover, the presence of efflux transporters (i.e. P-glycoproteins) in BBB and CBB also provides additional obstacles for drug delivery to the brain. Hence the next generation therapeutics for stroke therapy is expected to cross the BBB effectively. In brief, the clinical trials and reports obviously show an urgent need to apply the methods for swiftly crossing the physiological barriers and targeting site-specific administration of multiple curative agents at a wide range of time points to the management of IS.

## Crossing the BBB during IS

The BBB is one of the most vital component between the nervous system and rest part of the body, which protects the brain from harmful stimulation. BBB permeability is controlled by tight junction protein complexes (i.e., occludin, junctional adhesion molecules, claudins, membrane associated degrading enzymes and membrane-associated guanylate kinase-like proteins), which limits the paracellular diffusion between brain capillary endothelial cells (BCECs). Although stroke can destroy this barrier and lead to increased vascular permeability, the BBB maintains brain homeostasis [55]. It is reported that the BBB will be damaged to a certain extent in a short time during IS, allowing some endogenous substances (such as water and immune cells) or exogenous substances to enter the damaged brain tissue [56]. However, from the perspective of IS theranostic, the distribution of drugs to the site of stroke still needs to overcome the BBB penetration. As a new drug delivery technology developed in recent years, the nano-particulates drug delivery systems have provided an opportunity for the diagnosis and treatment of brain diseases.

Although passive delivery may facilitate the transport of nanocarriers across compromised BBB to some extent, lower dose and poor distribution of nanocarriers into the brain still remain great challenges [57]. Another attractive strategy to promote the nanocarriers across BBB is receptor-mediated transcytosis, which is facilitated by displaying high affinity ligands on the surface of nanocarriers [58]. This active transport, including adsorptive mediated and receptor-mediated transcytosis, has been widely developed to enhance selective targeting and reach the intracellular compartment after delivery. Firstly, the adsorption mediated transcytosis mechanism is based on surface functionalization (giving positive charge) of the nanocarriers and allows electrostatic interaction with BBB surface (negatively charged). Coating the nanocarriers with cationic compounds, such as cell-penetrating peptides and cationic protein, is also an alternative strategy [59]. The other promising method for nanocarriers across the BBB after the brain injury is receptor-mediated transcytosis. These receptor-mediated molecules, including transferrin receptors, low-density lipoprotein receptor, leptin receptor, insulin receptor and so on [60], could be functionalized to nanocarriers targeting receptors on the surface of BCECs.

## Nanocarriers in visualizing ischemic brain

A major requirement of IS treatment refers to visualizing the diseased site under great resolution and sensitive property for achieving precise diagnosis and continuously curative result monitoring. Numerous molecular imaging methods were employed to image the brain including MRI, positron emission spectroscopy (PET), computed tomography (CT), digital subtraction angiography (DSA), single photon-emission computed tomography (SPECT), ultrasound and optical imaging [61, 62]. Benefiting from the development of molecular biology, molecular imaging based on nanoplatforms has now emerged to become an interdisciplinary field. Using a molecularly targeted nanoplatform exhibits considerable merits in contrast to traditional approaches. First, nanotechnology dramatically facilitates a signal amplification since many imaging labels or combinations of labels in terms of various imaging modalities are attached to merely one single nanocarrier. Second, the multiple targeting ligands on the nanosystems are capable of endowing optimized binding affinity, specificity and targeting efficiency, thereby making it a reality to extend the lifetime in vivo, enhancing target-specific accumulation and bypassing the BBB presented in stroke under nanocarriers [63].

**Fig. 2:**
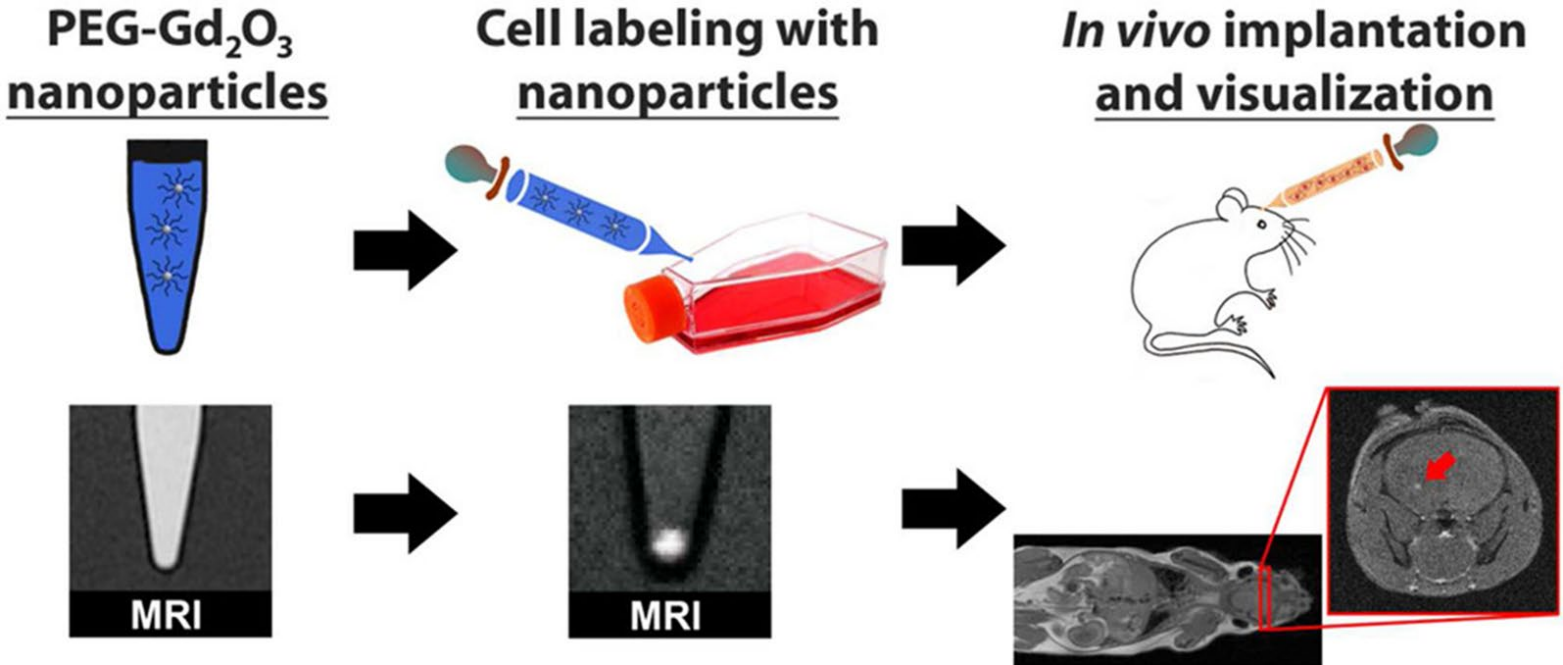
Design of PEGylated ultrasmall gadolinium oxide nanoparticles. Reproduced with permission from [69]. Copyright 2012, American Chemical Society

MRI refers to a non-invasive method for wide use to generate brain images in the absence of ionizing radiations, thereby presenting specific anatomical data and exhibiting the ability to distinguish normal brain tissue from infarct sites. Nevertheless, the signal intensity and sensitivity of MRI are generally lower as one medical diagnostic device. To tackle down the mentioned drawbacks, nanoparticles of paramagnetic molecules including magnetic nanoparticles (MNPs) were introduced as contrast enhancers. For instance, an investigation conducted by Ito A er al. reported that MNPs conjugated with antibodies were capable of exhibiting multiple-functionality as a selective biology-based recognizer of target molecules, as well as a MRI contrast enhancement agent [64]. It was indicated that combining the ferumoxide labelling (MRI contrast agent) with magnetic fields could facilitate targeted delivery of stem cells in ischemic brain injury [65]. To regulate the distribution, half-life and localization properties exhibited by MRI contrast agents, considerable classes of nanoparticles (NPs) encapsulated contrast agents were explored from the perspective of cerebral ischemia visualization [66]. Yu et al. proposed to employ lipid-encapsulated perflurocarbon nanoparticles as the positive MRI contrast agent Gd-DTPA complex [67]. The mentioned result revealed the potential for sensitive and specific detection of microthrombin that form on the intimal surface of unstable atherosclerotic plaques [68]. For the visualization of the collaterals during acute IS, an integrin α_v_β_3_-specific Fe_3_O_4_ nano-scale probe with surface modified by RGD tripeptide was prepared. The RGD peptide could be a particular recognition motif of integrin receptors, facilitating Fe_3_O_4_ nano-scale probe localization on collateral vessels and achieving MRI visualizing. Likewise, the PEGylated ultrasmall paramagnetic Gd_2_O_3_ nanoparticles with diameter of 5 nm were adopted to achieve cell labeling and tracking in the brain with MRI (Fig. 2) [69]. Furthermore, Varallyay CG et al. drew the comparison of the imaging efficiency exhibited by a carbohydrate-wrapped iron oxide nanoparticle, ferumoxytol and the gadoteridol. All of the mentioned particles were applied to determine the cerebral blood volume through the generation of images with steady states in the brain, which reported that ferumoxytol could exhibit higher resolution as compared with the dynamic susceptibility contrast perfusion imaging of gadoteridol [70].

PET refers to a method to analyze brain functions with the use of a radioactive tracer, thereby allowing for metabolic marker mapping and physiology-related factor within the brain region [71]. As one invaluable approach for researching cerebral ischemia, it is constantly combined with MRI and CT to achieve better imaging purpose. For instance, Glaus C et al. have developed a newly developed nanoparticle-based dual-modality PET/MRI contrast agent. This study labelled the nanoparticles comprising a superparamagnetic iron oxide (SPIO) core with ^64^Cu and then coated it with PEGylated phospholipids. Biodistribution and in vivo PET/CT imaging researches on the probes indicated high initial blood retention with moderate liver uptake, thereby enabling them to be attractive contrast agents for disease researches [72]. Likewise, ^18^ F-modified polyglucose nanoparticles that exhibited high affinity for macrophages in MRI and PET imaging of ischemic animals have been reported. The mentioned data suggest that macroflor PET/MRI could be a clinical device for the non-invasive monitoring of macrophage biology [73].

**Fig. 3:**
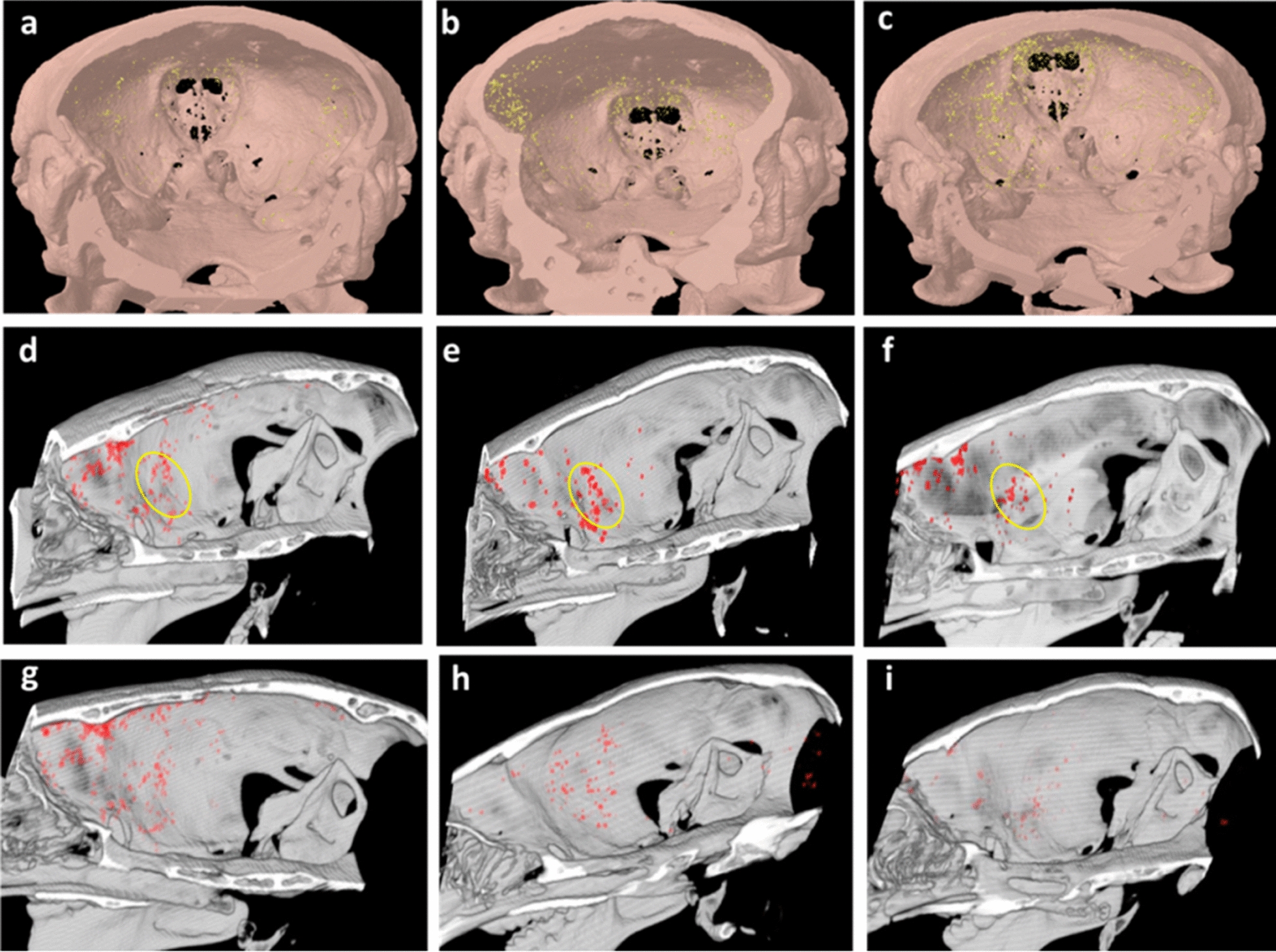
In vivo CT imaging of exosomes after acute striatal stroke in a mouse model. **a**–**f** Ischemic brain: coronal and sagittal three-dimensional (3D) volume rendering views of typical brain, wherein ischemic insult was induced in the striatum in the right hemisphere: **a**, **d** 1 h, **b**, **e** 3 h, and **c**, **f** 24 h post-exosome administration (the ischemic region is demarcated in yellow circle). Exosomes can be seen to migrate and accumulate at the ischemic region at 3 h. **g**–**i** Control brain: sagittal 3D volume rendering views of control mouse brain: **g** 1 h, **h** 3 h, and **i** 24 h post-exosome administration. Exosomes penetrated the brain but did not specifically accumulate in any region and were cleared from the brain over 24 h post-administration. Reproduced with permission from [81]. Copyright 2017, American Chemical Society

CT refers to a highly powerful non-invasive diagnostic imaging method in modern medicine, to be integrated and processed to generate tomographic images of the brain. Compared with MRI, CT is not sensitive to the differentiation between ischemic penumbral and infarct core region [74]. According to the general information, CT angiography (CTA) exhibits prominent performance in large vessel occlusion and perfusion imaging, and CT can present an initial idea about the hypoperfusion and hypometabolism of the microvasculature system. Under cerebral ischemia, iodinated molecules have a relatively short circulation time in vivo acting as CT contrast agents in clinical practice, which noticeably restrict their applications in target-specific imaging and angiography [75]. Moreover, Xenon gas has gained an extensive application to achieve contrast enhancement in ischemic penumbra due to its easy diffusion in the tissues, the capability to cross the BBB, the inert nature and biocompatibility. However, its anesthetic properties exerted adverse effects, thereby requiring alternate contrast agents [76]. Thus, biomedical researchers have exerted tremendous efforts to address the mentioned issues. For instance, a study reported that the Au nanocarriers grown in the PEGylated dendrimers could be used as CT contrast agents, which have been proved to achieve a blood pool imaging better than commercial iodine agents [77]. PEGylated BaHoF_5_ nanoprobes with a mean diameter of 7 nm were also developed and employed as a contrast agent for CT angiography (CT-A) and CT perfusion (CT-P) imaging. This nanoprobe has unique advantages over traditional iodinated CT agents, such as much lower dosage needed, metabolism primarily through liver and higher imaging efficiency at different voltages [78]. In addition, polymeric nanosystems incorporating with gold nanoparticles were investigated extensively because of their versatile features which can be modified with other therapeutic, targeting or imaging moieties [79, 80]. Apart from polymeric carriers, glucose-coated exosomes labeled with gold nanoparticles were exploited for cerebral stroke imaging analysis (Fig. 3), whose results indicated that nasal administration showed better particle accumulation than intravenous administration in ischemic area [81]. Accordingly, it is evident that nano-delivery of contrast agents can better visualize the penumbra in brain.

**Fig. 4:**
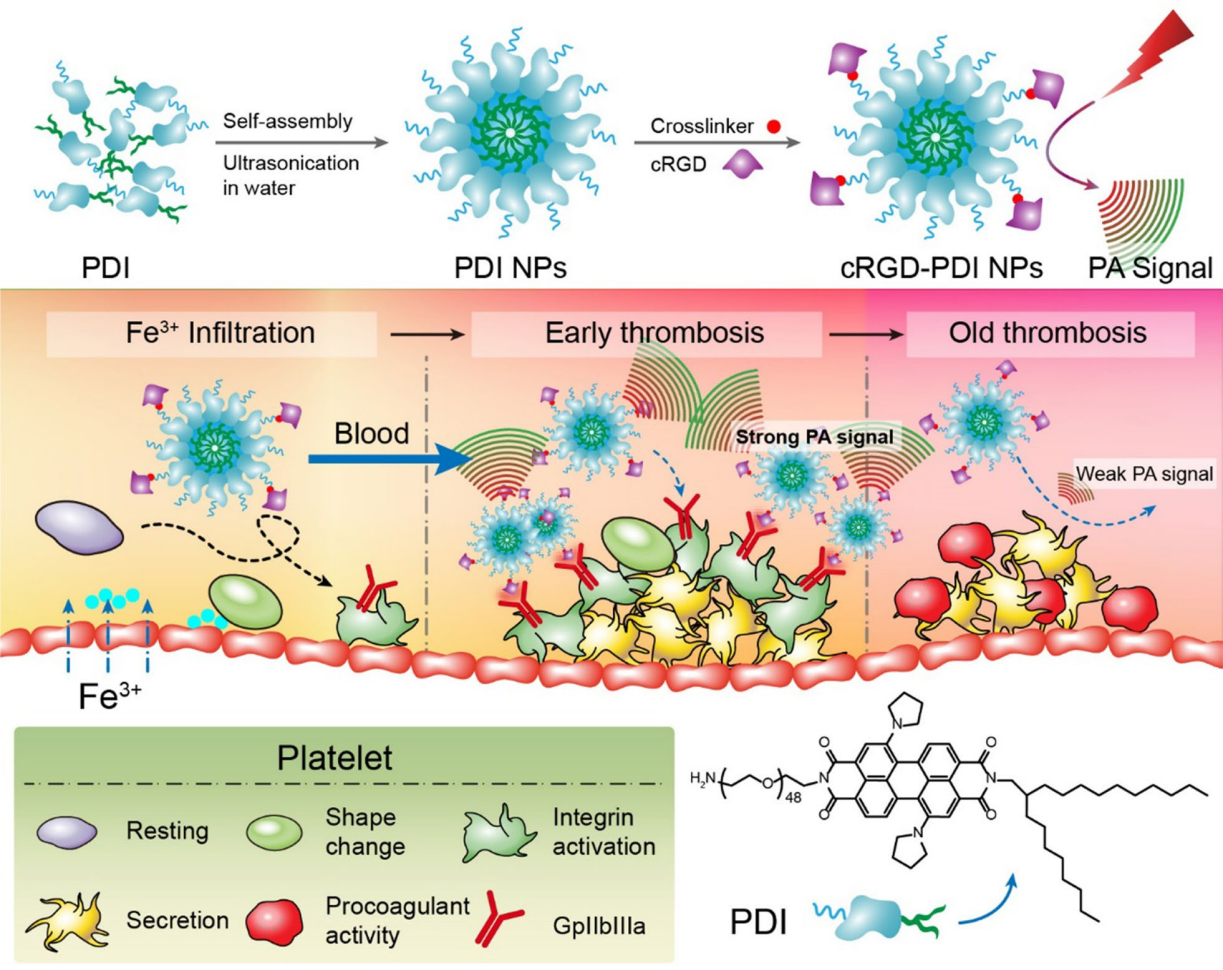
Design schematic illustration of the preparation of cRGD-PDI NPs and its mechanism for specifically lightening early thrombus by PAI. For PAI, 5% FeCl3 was applied to the jugular vein and diffused through the vessel wall, resulting in the exposure of basement membrane components to circulating blood cells. Platelets were then activated to deform, adhere, and aggregate together to form early thrombus during the vascular intima injury. The initial resting integrin GPIIb/IIIa on the platelets transformed into a high-affinity state in early thrombus and finally became a low-affinity state when the early thrombus grew into an old thrombus. cRGD-PDI NPs can target GPIIb/IIIa in early thrombus while inefficiently in old thrombus, resulting in selectively lightening early thrombus by PAI. Reproduced with permission from[84]. Copyright 2017, American Chemical Society

Ultrasound images are achieved by adopting a small probe as a transducer and gel placed directly on the skin to image the blockage or defects in blood vessels. It creates images of the inside of the body with the use of sound waves known as echogenicity. Blood vessels are known to exhibit poor echogenicity and hardly reflect the ultrasound waves even under a high intensity. As a result, ultrasound contrast enhancers are introduced into the system, which are usually aerated microbubbles exhibiting robust echogenic properties made of albumin, lipids or synthetic polymers [82, 83]. Over the past few years, surface modification with targeting ligands has enabled the microbubble system to cluster at specific sites, thereby presenting higher resolution images. Although ultrasonography does possess several advantages in contrast to MRI and CT, its application has been dramatically limited in brain imaging, primarily resulting from the difficulty of penetrating the cranium. To overcome the difficulty, organic semi-conductors perylene tetracarboxylic diimide (PDI) nanoparticles coupled with cyclic RGD performed well in generating photoacoustic images of the blood clots, providing timely monitoring of thrombosis obstruction in blood vessels and thrombolysis effect (Fig. 4) [84]. Several researches are currently underway on the potential of nanocarriers worldwide, including exogenous nanoparticle-based agents for PDI applications covering contrast agents on the basis of gold particles, carbon nanotubes and copper-encapsulated compounds [85]. All the mentioned innovations are presently in their initial phases and more studies in such a direction are urgently required to achieve clinical translation of this imaging mode.

So far, several clinical trials have been conducted for imaging of IS while very few of them involve nanocarriers. The benefits of nano-based interventions in the context of IS imaging have been clearly demonstrated in many in vitro and pre-clinical studies. Considering that many inorganic materials do not naturally exist in the body, fully evaluating the biodistribution and toxicity after systemic administration is urgent needed. This is expected to initiate more clinical trials involving nanoparticle-mediated imaging strategies for IS in the future (Table 2).

**Table 2:** Nanocarriers applied in molecular imaging methods to visualize the ischemic brain

Molecular imaging methods	Shortcomings before enhancement	Improved methods for applying nanocarriers	Results	Refs.
Magnetic resonance imaging (MRI)	Low signal intensity and sensitivity	MNPs conjugated with antibodies	Become both a MRI contrast enhancement agent and a selective biological recognizer of target molecules	[64]
		Lipid-encapsulated perflurocarbon NPs	Detect the microthrombin that form on the intimal surfaces of unstable atherosclerotic plaque sensitively and specifically	[67]
		An integrin α_v_β_3_-specific Fe_3_O_4_ nanoprobe modified with RGD tripeptide	Visualize the collaterals during acute IS	[68]
		PEGylated ultrasma paramagnetic Gd2O3 NPs	Label and track cells in brain	[69]
Positron emission spectroscopy (PET)	Always used together with MRI and CT	NPs consisted of a SPIO core labelled with ^64^Cu and coated with PEGylated phospholipids	Show high initial blood retention with moderate liver uptake	[72]
		^18^F-modified polyglucose NPs	Exhibit high affinity for macrophages	[73]
Computed tomography (CT)	Not sensitive within the differentiation of penumbral and infarct core region	Au NPs grown in the PEGylated dendrimer	Achieve a blood pool imaging better than a commercial iodine agent	[77]
		PEGylated BaHoF_5_ nanoprobes	Much lower dosage required, main metabolism through liver and better imaging efficiency at different voltages	[78]
		Exosome-labeled with glucose coated gold nanoparticles	Exhibit better accumulation of particles in the ischemic region	[81]
Ultrasonography	Difficult to penetrate the cranium	PDI NPs conjugated with cRGD	Monitor the obstructive degree of thrombus and the thrombolysis effect in time	[84]

## The application of nanocarriers in IS treatment

**Fig. 5:**
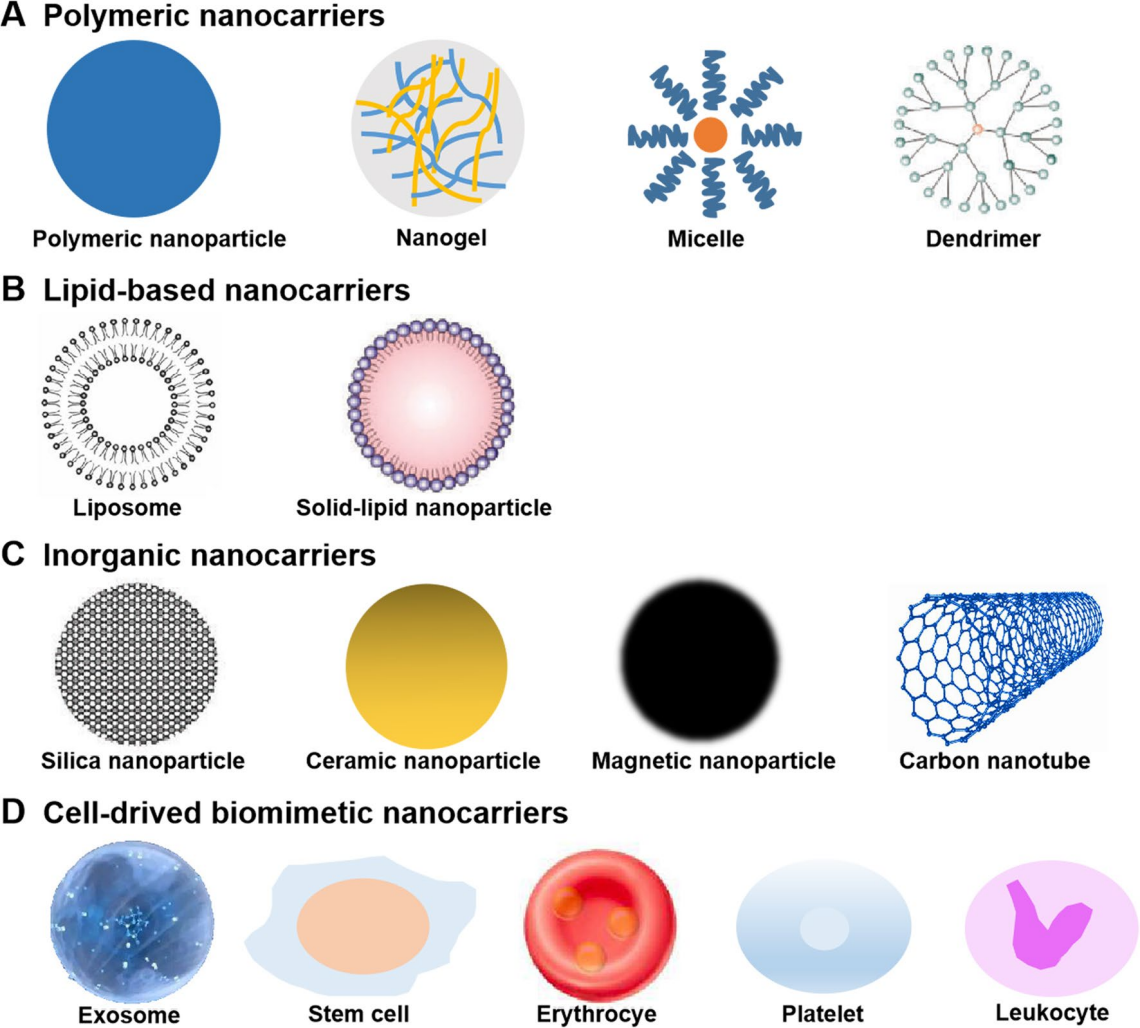
Principle scheme of nanocarriers for the management of IS. Each type of nanocarriers was discussed in the manuscript

Apoptosis or cell injury within ischemic core occurs fast, causing the difficult protection of such a region by conventional approach. Common drawbacks that limit traditional drug treatment consist of the physiological barriers, fast metabolism or degrading process, as well as unsatisfactory distributing profile. The nano drug delivery systems are capable of deriving from material exhibiting organic backgrounds or artificial features. As a drug-delivery system with high prospect in treating brain disorder, the nanocarriers exhibit benefits in preventing drug degradation, improving pharmacokinetic profiles and neurovascular unit access. Curative agent is typically encapsulated, entrapped, adsorbed or chemically attached on the surface of nanocarriers [86]. The nanocarriers can also cross the BBB through adsorptive-mediated or receptor-mediated transcytosis channels on the basis of positive charge display or ligands surface conjugation [87, 88]. Herein, we summarize several promising nanocarriers for the management of IS (Table 3; Fig. 5).

### Polymer based nanocarriers

The polymer based nanocarriers have a wide range of applications in the IS delivery due to the good biodegradability and abundant functional groups, which make it easy for targeted functional modification. The most common polymer based nanocarriers include polymeric nanoparticles, polymeric micelles, nanogel, dendrimer. The detailed introduction will be listed below.

#### Polymeric nanoparticles

The polymers include not only the synthetic biodegradable polymers including poly(lactic acid) (PLA), poly(L-lactide-co-glycolide) (PLGA), polycaprolactone and polyethylene glycol (PEG) PLGA, but the natural macromolecular systems consisting of chitosan, polysaccharide, gelatin and starch. In recent years, polymer nanoparticles have been broadly used as the delivery systems for the treatment of IS. PLGA is widely employed among the biodegradable polymers mentioned above. It is a biodegradable polymer under hydrolysable ester links approved by FDA. The gradual hydrolyzing process of PLGA leads to a reduction in molecule weight and formation of voids in polymer chains, thereby facilitating the sustained-release of the drug. Mdzinarishvili et al. conducted the application of glutathione-coated PLGA-b-PEG nanoparticles to load thyroid hormones (T3), which has been proven for protecting the brain from ischemic injury through glutathione transporter on the BBB [89]. Furthermore, gene silencing represents as a newly developed curative strategy for the treatment of IS. Certain siRNAs always realized their curative potential with the help of polymeric nanoparticles because of their low stability against nuclease reduction and lack of capability to permeate the BBB [90, 91].

**Fig. 6:**
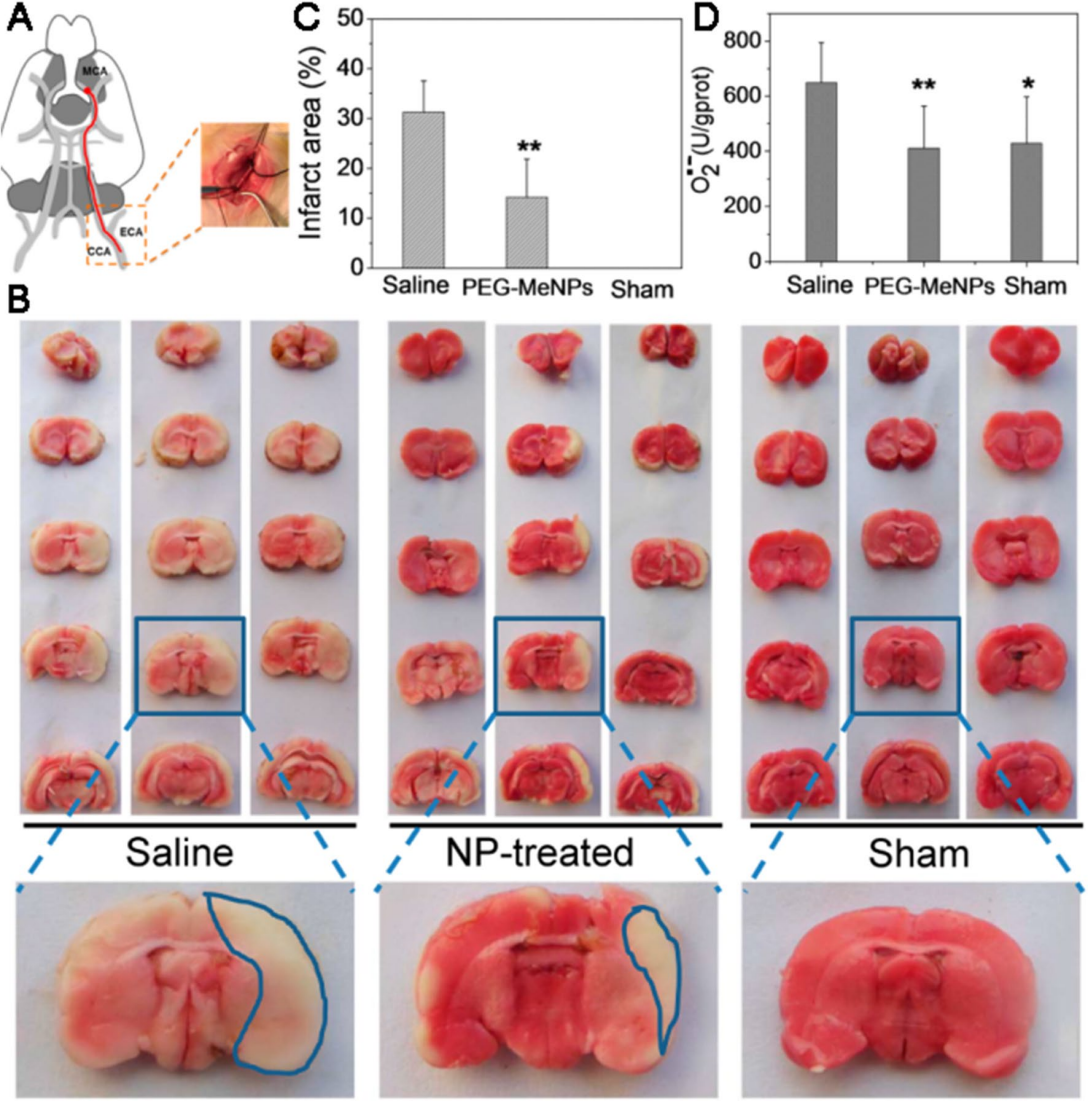
**A** Scheme presentation of the IS model. **B** Typical pictures of TTC-stained brain slices in a range of groups. The relative **C** infarct regions and **D** O2•-levels brain tissue in 3 groups (*p < 0.05 and **p < 0.01 vs. saline control). Reproduced with permission from [96]. Copyright 2017, American Chemical Society

Besides synthetic polymer, natural biodegradable polymers (including chitosan, melanin and gelatin) are also commonly used to prepare nanoparticles. For instance, Vemisci et al. have shown that bFGF or z-DEVD-FMK encapsulation in chitosan nanoparticles excited large quantities of nanoparticles crossing the BBB [92]. Another research group synthesized chitosan-conjugated NIPAAM (N-isopropylacrylamide) nanoparticles coated with Tween 80 through free radical polymerization. This study has demonstrated that the nanoparticles were sufficient to carry large amounts of drugs across the BBB and could play a significant neuroprotective effect even at a very low concentration [93]. The melanin is famous as a free radical scavenger, covering functional groups including imine, amine as well as catechol, which has been proved to detoxify several reactive oxygen species and nitrogen species (RONS) [94, 95]. Liu Y et al. developed a natural melanin nanoparticle (MeNPs) with higher antioxidative effectiveness and safety (Fig. 6) [96]. Likewise, the gelatin nanoparticles have also been developed as drug carriers for the management of IS [97], in which osteopontin, an endogenous protein with neuroprotective effects, is a payload. Though polymeric nanoparticles are good options for drug delivery system, they have certain limitations that include high cost, sophisticated skills required in manufacturing process and potential toxicity of organic solvents introduced in the preparation [98, 99]. We firmly considered that the polymeric nanoparticles could have more promising applications in the near future only if their preparation conditions and properties can be optimized more specifically.

#### Polymeric micelles

Polymeric micelles refer to newly developed nano-scale systems employed for incorporating charged molecules, acting as attractive options to deliver drugs for spontaneously self-assembling property. This nano-micelles are established under the reaction in which double hydrophilic block copolymers covering ionic and non-ionic blocks are reacting with macro-scale molecules pertaining to opposite charge (e.g., oligonucleotide, plasmid DNA and protein) [100, 101]. For instance, the polymeric micelles were explored to deliver DNA molecules efficiently in vitro and in vivo [102, 103]. Micelles formed by PEG-b-poly(methylstyrene) block copolymer could also become one potent nanocarrier to increase brain ischemia/reperfusion (I/R) injury pharmacotherapy, with unprecedented clinical benefits, and be used to deliver tumor necrosis factor-α (TNF-α), thus ameliorating oxidization stress injuries, inflammation-related activity and cell death in infarct sites [104]. Another study has obtained EDV-AM for delivering edaravone in ischemia of brain through active regulation of permeability of the BBB [105].

#### Dendrimers

**Fig. 7:**
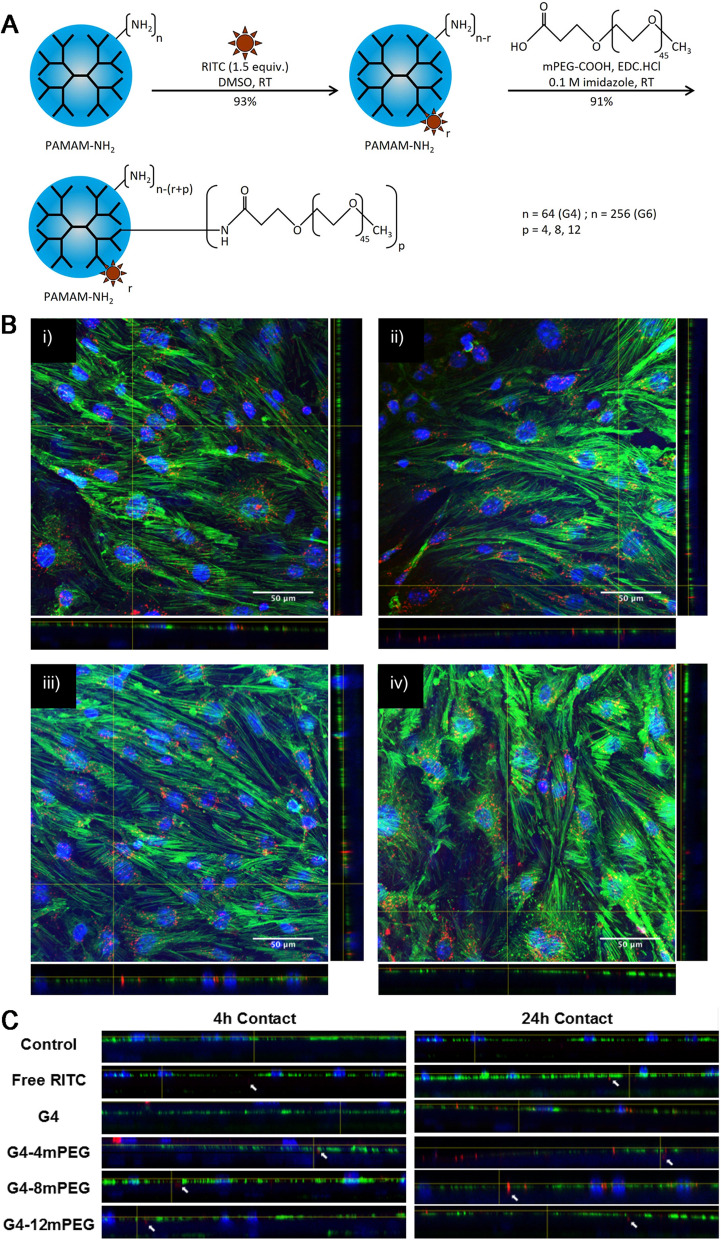
**A** Design of RITC-labelling and mPEG conjugation of PAMAM-NH_2_. **B** Fluorescent confocal microscopy images showing bEnd.3/rat primary astrocytes in vitro BBB model in contact with PEGylated G4 PAMAMRITC dendrimers max intensity Z projections and orthogonal views of the in vitro BBB model layers at 24 h, after 4 h contact with (i) G4 PAMAMRITC, (ii) G4 PAMAMRITC-4mPEG, (iii) G4 PAMAMRITC-8mPEG and (iv) G4 PAMAMRITC-12mPEG is shown in red, the cell nuclei are stained with Hoechst (blue) and F-actin was stained with Alexa Fluor® 488 phalloidin. **C** Horizontal orthogonal views of the in vitro BBB model at 4 and 24 h after 4 h contact with PAMAMRITC conjugates or free RITC. Reproduced with permission from [110]. Copyright 2018, Elsevier

The dendrimers, i.e., a type of highly branched and monodispersed macrocyclic polymer micelles, cover numerous features including a core with attached branch, with branch shell around the core, i.e., a surface of multivalence, the internal shell and the inner core covering terminal function-related group and repeating unit [106]. In particular, dendrimers exhibit consistent architecture with micelle, the micelle carriers established from dendrimers, thereby enabling them to be more appropriate for delivery of drug and gene [107]. The polyamido amine (PAMAM) has become the most broadly applied in a wide range of biomedical uses in the dendrimers system [108]. The 4th- and 5th-generation PAMAM dendrimers are suggested to be capable of binding DNA and enhancing the delivery in the cell [109]. In a recent research, the biocompatibility of the PAMAM dendrimer had noticeable improvement through PEGylation as functionalization degree (Fig. 7). The PEGylated PAMAM dendrimers failed to impact the BBB integrity within in vitro model, and nor did they show cytotoxicity within in vitro hypoxia model based on deprivation of oxygen and glucose [110]. In addition, gene transferring across the brain capillary cells was suggested with the use of a transferrin-conjugated PEG-modified PAMAM dendrimer [111]. Furthermore, Kim I D et al. adopted PAMAM dendrimers as a nanocarrier to deliver siRNA in neuron cell, and analyzed siRNA delivering efficiency through e-PAM-R (arginine ester of PAMAM) in main cortical culture as well as brains of mice. The e-PAM-R/siRNA complexes indicated great efficiency of transfection and low cytotoxicity inside primary cortical culture, thereby revealing that e-PAM-R, a newly developed gene carrier with biodegradability, offered effective approach, i.e., to transfect siRNA in major neuronal cell and the brain as well as to perform gene knockdown under the mediation of siRNA [112]. Keeping in view of these specialized features of dendrimers, a dendrimers-based nano-agonists (NAs) was developed for targeted delivery of SOD to ischemic brain regions, enhancing the neuroprotectant uptake by specifically increasing BBB permeability in brain [113]. However, dendrimers micelles showed probable biological toxicity (including cytotoxicity and hematotoxicity), thereby delaying the clinical uses [114].

#### Nano-hydrogels

Nano-hydrogels are hydrophilic 3D polymer network under broad applications within drug delivery and tissue engineering, capable of maintaining a certain three-dimensional shape and loading a large number of charged molecules [115]. Nano-hydrogels are receiving investigation of CNS delivery of a wide range of low-molecular-mass compounds and biomacromolecules including siRNA, DNA and oligonucleotides [116, 117]. Studies have demonstrated that the oligonucleotides loaded by nano-hydrogels system could transport in the brain well, which was mediated by the transcytosis of BMEC [117]. Vascular endothelial growth factor (VEGF) refers to a potent proangiogenic peptide, i.e., a possible neuroprotective method for cerebral stroke. However, ineffective penetration across the brain parenchyma and low half-life noticeably hinder the use of VEGF when treated systemically [118]. For instance, Emerich DF et al. adopted the identical sustained-release delivery systems on the basis of injectable hydrogel (alginate) for evaluating VEGF effects on middle cerebral artery (MCA) function and anatomical transient occlusion in mice [119]. As proved in this study, VEGF, under the direct delivery from hydrogel to brain, was capable of inducing noticeable function-related and structure-related protective effects against ischemic injury in stroke rat model. When noticeable progress has been achieved within the development and the optimization of nano-hydrogel formulations, difficulties in biocompatibility and in vivo toxicity continue to enhance the clinical applicability for drug delivery.

### Lipid-based nanocarriers

#### Liposomes

Liposomes refer to vesicular carriers receiving the self-assembly, under the formation of amphipathic lipids through the phospholipids and cholesterol mixing within various ratios [88, 120], is a type of nano-scale structure developed for drug delivery and are currently the nanocarriers with high frequency in clinic and clinical tests for their poor toxicity, prominent drug loading capacity and high biocompatibility [121, 122]. Thus far, obvious progress has been made in studies on lipid-based methods to manage IS. For instance, the citicoline (cytidine-5-diphosphocholine), which has been reported to be neuroprotective under cerebral ischemia/reperfusion injury [123, 124], receives the rapid metabolization from the liver, triggering the incapability to reach the brain if treated under the systemic circulation [125]. Citicoline encapsulated in liposomes has been proven to reduce hepatic hydrolysis and noticeably reduced infarction in contrast with the identical free citicoline’s dose regime [126, 127]. To inhibit neutrophils’ infiltration, shimbo D et al. reported that post-ischemic intra-arterial infusion of LEH was capable of reducing ischemia/reperfusion (I/R) injury based on the reduction of MMP-9 effect, most likely generated from neutrophils [128]. Very recently, the cRGD liposomes that encapsulated urokinase have been demonstrated to exhibit greater thrombolytic efficacy [129].

**Fig. 8:**
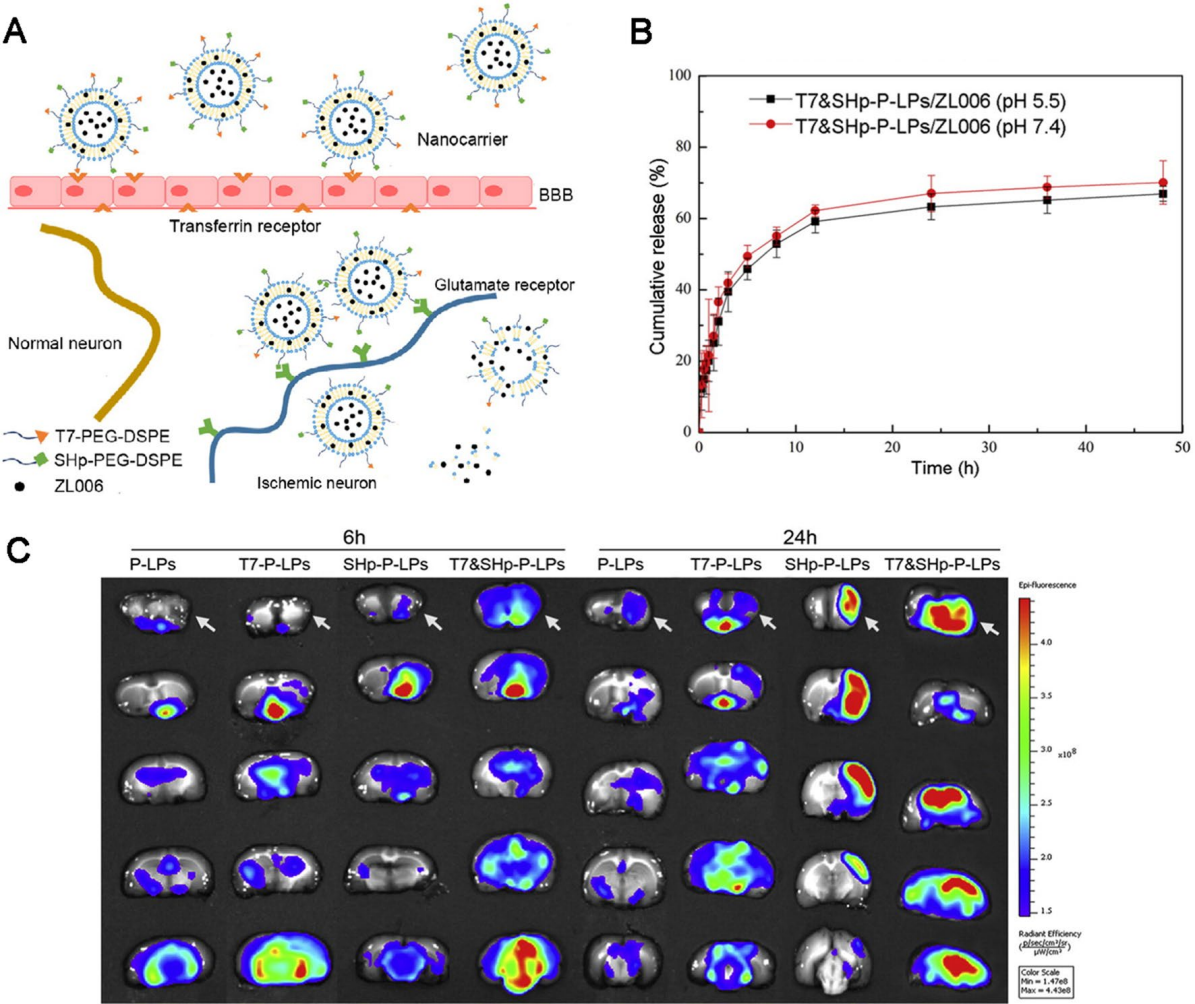
Schematic design and characterization of T7&SHp-P-LPs. **A** Designs for ZL006 loaded T7 and PEGylated dual targeted nanocarrier under the SHp conjugation in terms of focal cerebral ischemia treatment through transcytosis under the mediation of transferrin receptor and endocytosis under the mediation of glutamate receptor. **B** ZL006 release profiles from T7&SHp-P-LPs in PBS (pH 5.5) and PBS (pH 7.4) containing 0.1% Tween-80 (n = 3). **C** Ex vivo fluorescent image of DiR-labeled P-LPs, T7-P-LPs, SHp-P-LPs and T7&SHp-P-LPs in the ischemic brain at 6 and 24 h. Arrow: ischemic cerebral hemisphere. Reproduced with permission from [140]. Copyright 2016, Elsevier

A critical defect of liposomal systems refers to their narrow circulation time in the body. PEGylation of drug-loaded liposomes exhibits particular effectiveness to improve their longevity within systemic circulation [130]. Recently, PEGylated liposomes have been proven to have the ability to boost drug activity in vivo, including EPO [131], minocycline [132], FK506 [133] and citicoline [134]. Moreover, PEGylated liposomes were adopted to regulate the xenon release in the brain, thereby achieving physical targeting based on outer ultrasound application [135]. PEGylated liposomes can receive the in-depth modification with transferrin or other target peptides for improving their ability of active targeting delivery to affected regions in the brain [136, 137]. For instance, ZL006, a compound selectively blocking NO synthase coupling of neurons attributed to by ischemia, exhibits potent neuroprotective activity in vitro and is capable of ameliorating focal cerebral ischemic impairment in MCAO reperfusion mice [138]. Our group developed liposomes under the modification of BBB targeting T7 peptide with a HAIYPRH sequence especially interacting with transferrin for enhancing the delivery of ZL006 across BBB [139]. Furthermore, a ZL006-loaded dual targeted nanocarrier (T7&SHp-P-LPs/ZL006) was designed by complying with PEGylated lipids under the conjugation of SHp and T7 peptide in terms of BBB penetration and ischemia region targeting, respectively (Fig. 8), thereby revealing that the ZL006-loaded dual targeting liposome was capable of well targeting cerebral ischemia/reperfusion injury lesion, noticeably reducing the infarct volume and improving neurology deficits in MCAO-induced cerebral ischemia/reperfusion injury [140].

Despite the advantages of liposomes, currently there are still no liposome-based nanocarriers available in clinical for the treatment of IS. As a drug delivery, liposomes have been broadly proved to improve pharmacokinetics and biodistribution. Yet no currently marketed liposomal curative agents have exhibited an overall survival benefit when directly compared with the traditional drug for IS, which probably result from the lipid instability, drug leakage and insufficient targeting at the target site [141, 142].

#### Solid lipid nanoparticles (SLNs)

SLNs are also stable lipid-based nanocarriers featuring one core of solid hydrophobic lipid for drug encapsulating process, comprising biocompatible lipid including fatty acid and triglyceride [58, 143]. SLNs are preferred more over polymeric nanoparticles as impacted by considerable merits including small intrinsic cytotoxicity, physical stable property, labile drug shielding from degradation, regulated release providing them to probably act as drug delivery for IS treatment. For instance, the authors assessed curcumin loaded solid lipid nanoparticles in mice with cerebral ischemia, and the result indicated an enhancement of 52% suppression of acetylcholinesterase level and 90% in cognition [144]. Selective overexpression of Fas ligands has been reported in ischemic region cells. Lu et al. reported that the PEGylated-liposomal nanoparticles decorated by Fas ligand antibodies could well transport across the blood–brain barrier along with less inflammation-related responses [145]. The neuroprotective agent (baicalin) received the encapsulation in SLNs with an average size of 100 nm as well as negative ζ-potential of -50 mV, and the results indicated that the increase of baicalin in the brain stem and cerebral cortex was greater than that of free baicalin [146]. PEGylated cationic SLNs under the conjugation with an OX26 antibody enhanced uptake of baicalin across the BBB, while elevated the bioavailability of baicalin in cerebrospinal fluid of mice with cerebral ischemia-reperfusion injuries [147].

### Inorganic nanocarriers

Besides polymeric and lipid-based carriers, the inorganic nano-scale materials, including silica, alumina, metals, and carbon, have shown great potential in the treatment of IS [148]. Inorganic nanocarriers provide advantages over sizes and shapes controlling of polymeric nanoparticles and simple preparing process and functionalizing process. Notably, inorganic nanocarriers can more easily be tracked based on microscopy methods (including transmission electron microscope (TEM), magnetic resonance imaging (MRI)) or analytic methods. The synthesis of silica nanocarriers is able to be achieved with the use of calcium phosphate by employing a hollow core for physically entrapping payload. Furthermore, silica core nanocarriers under the coating by employing thin gold shell are applied for producing a wide range of shapes including rod, shell, and sphere. Ceramic carried with silica are explored as well to deliver curative drugs for cerebral ischemia and related complications [149]. Magnetic nanocarriers act as contrast agents by combining with iron oxide in MRI, PEG or silica are always coated for diagnostic applications of IS [150, 151]. Gold nanoparticles (Au-NPs), commonly known as gold colloid, are also widely used for drug delivery or bioanalysis. For example, the Au-NPs were designed for investigating the potential effects on rat cortical neurons exposed to oxygen–glucose deprivation/reperfusion (OGD/R), suggesting that Au-NPs may be potential therapeutic agents for ischemic stroke [152]. Aptamers are single-stranded oligonucleotides, DNA or RNA, with the ability to bind to inorganic molecules (especially gold nanoparticles), offer great promise for applications of IS treatment [153, 154]. Chen et al. reported a silica coated magnetic nanoparticles (SiO_2_-MNP) conjugated with tPA, proven to be magnetically guided to increase in a thrombolysis model ex vivo. The SiO_2_-MNP developed here exited a great future to be the carrier of magnetic targeting drug for improving clinically related thrombolytic therapies [155]. Carbon nano-scale tubes (CNTs), defined as cylindrical nano-scale materials composed of a continuous hexagonal mesh of carbon atoms, refer to type of inorganic materials being utilized as drug carriers for stroke therapy [156, 157]. The mentioned nano-scale materials have been frequently adopted to be drug carriers [158, 159] as impacted by their cage-like architecture, low size and surface functional modification capability [160, 161]. For instance, Costa P M et al. reported that the amino-modified CNTs could be internalized in neurons through the observation of the transmission electron microscope. It has been reported that the number of cell apoptosis in the brain region decreased noticeably when administrated with the amino-modified CNTs [162]. Some authors have reported the two different nanoparticle forms of vitamin C, comprising silica-coated Au nanoparticles or polymer micelle on the basis of lipophilic polyaspartic acid. This result indicated that vitamin C could protect cell against oxidative stress under micromolar concentration [163].

As reported from recently conducted researches, several specific inorganic nanocarriers themselves can serve as ROS scavengers in the course of cerebral ischemia, especially after reperfusion. Platinum nanoparticles (nPts) exhibited antioxidant activity through the process to scavenge hydrogen peroxide and superoxide anions when tested in vivo [164]. Cerium oxide nanoparticles show the identical ROS scavenging property in the course of oxidative variation of cerium (reducing process-oxidizing process) as well, thereby exerting a significant neuroprotective effect for IS [165, 166]. The mentioned nanosystems become antioxidants through the free radical scavenging activity and down-regulation of RNS and ROS concentrations [167]. And the inorganic nanocarriers have efficiency at super-small scale (i.e., 4 nm), enabling it to be a prominent candidate against oxidative injury in the ischemia brain [166].

In the recent years, the newly developed integrated hybrid nanocarriers have been gaining attention including organic-inorganic hybrid nanocarriers and lipid-polymer hybrid nanoparticles. It is well known that the organic-inorganic hybrid nanocarriers have the properties of reducing initial drug burst and adjustable surface chemistry [168, 169]. Furthermore, the core-shell structure of lipid-polymer hybrid nanoparticles brings in great structure integrity, controlled drug release as well as extended shelf-life [170, 171]. Nevertheless, the inorganic nanocarriers also have shortcomings because their retention time and metabolic mode in the brain remain unclear, requiring further observation [172]. More importantly, present undesired toxicity of inorganic nanocarriers will limit their application in vivo (i.e., carbon nanotubes may contribute to oxygen radical formation as well as lipid peroxidation). All in all, though the mentioned systems have shown great prospects in vitro, there is an urgent need to report on the in vivo assessment of the carriers to assess their suitability as curative interventions.

### Cell-derived biomimetic nanocarriers

Biomimetic carriers from cells have presented novel option for drug delivery as impacted by the advantage of distinctive functions and prominent delivery mechanisms of endogenous cells. Camouflage by means of a natural cell or cell membrane has gained increasing attention, because it bypasses the recognition of nanocarriers by the mononeuclear phagocyte mechanism, thereby reducing immunogenicity and prolonging circulation [173].

#### Exosomes

Exosome refers to a type of vesicular carriers secreted by various cells, which has elicited great interest due to its unique characteristics of low immunogenicity, inherent stability, high delivery efficiency and capability to cross the BBB, to be utilized as a drug and gene carrier to treat IS. For instance, Yang et al. have reported that the modified exosomes fusing rabies virus glycoprotein (RVG) and exosomal protein lysosome-correlated membrane glycoprotein 2b (Lamp2b), could efficiently deliver miR-124 to the infarct site [174]. This RVG-exosomes can be employed therapeutically for the targeted delivery of gene drugs to the brain, thus having great potential for clinical applications. In a similar strategy, the exosomes conjugated with the c(RGDyK) peptide were applied as targeted drug delivery vehicles for cerebral ischemia treatment [175].

#### Stem cells

**Fig. 9:**
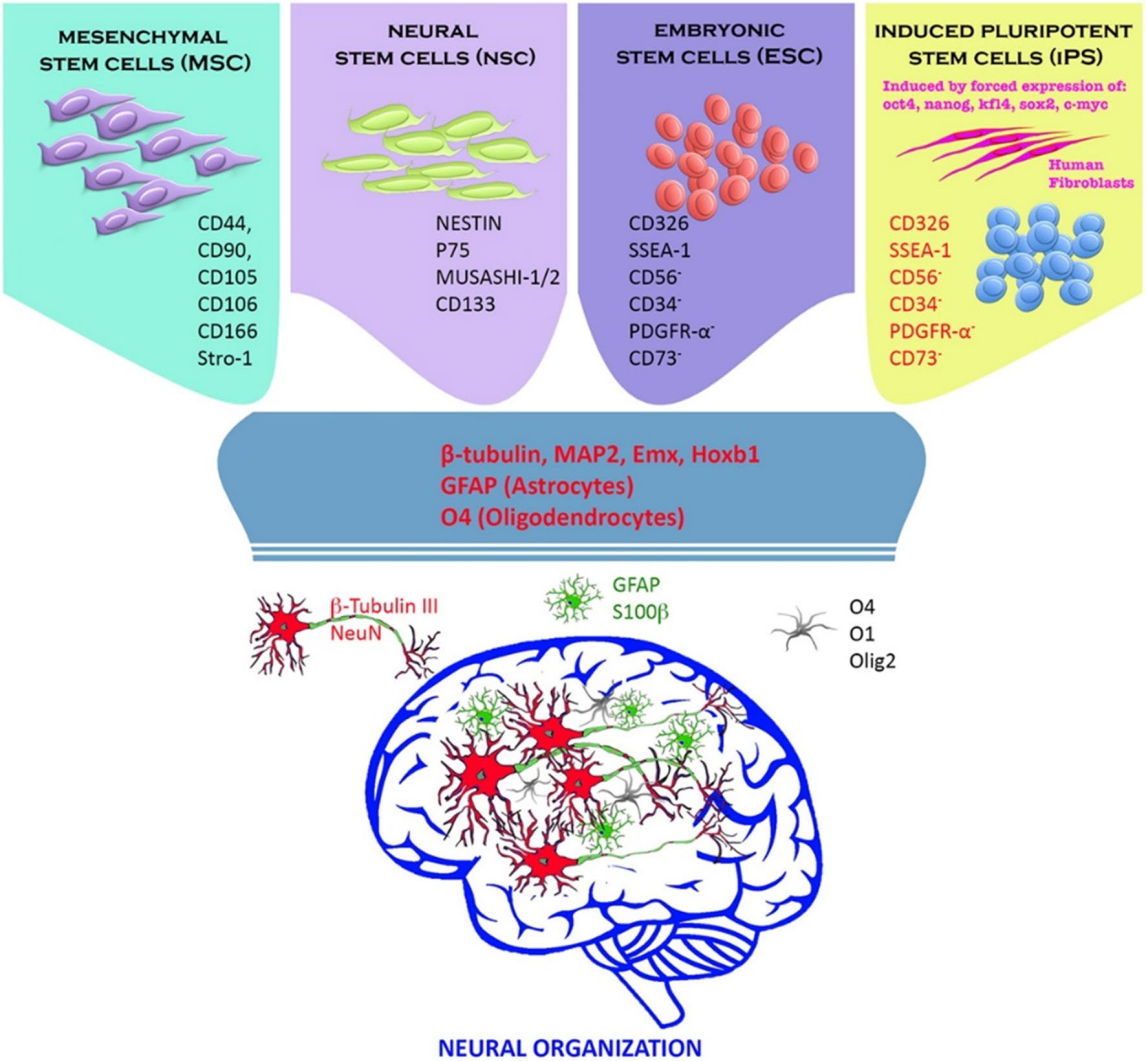
Stem cell and neural progenitor cell were applied for replacing death of neural tissue under a cerebral insult. Adult (mesenchymal and neural stem cell) and ESCs showed prominent capacity in differentiating the neural phenotype (astrocyte, oligodendrocyte, and neuron) in vitro and in vivo. Reproduced with permission from [179]. Copyright 2018, Frontiers Media S.A

Another attractive project in the context of cell-based stroke therapy is employing stem cell to remodel the function-related neurons lost as impacted by the ischemia/reperfusion injuries [176]. Various stem cell sources (e.g., neural stem cells (NSC) [177], bone marrow derived stem cells (BMDSC), mesenchymal stem cells (MSC) [176] and induced pluripotent stem cells (iPSC) 178 have been explored for IS. Thus far, some preclinical and clinical studies are underway to evaluate the curative efficacy of stem cells in the treatment against stroke and correlated complications (Fig. 9). As impacted by restricted time window in recorded clinical researches mostly concerned with adopting stem cell to be probable curative intervention of stroke, thus the in-depth clinical tests assessing the intervention efficacy based on a longer time window after cellular engraftments should be further carried out [179].

#### Erythrocytes

**Fig. 10:**
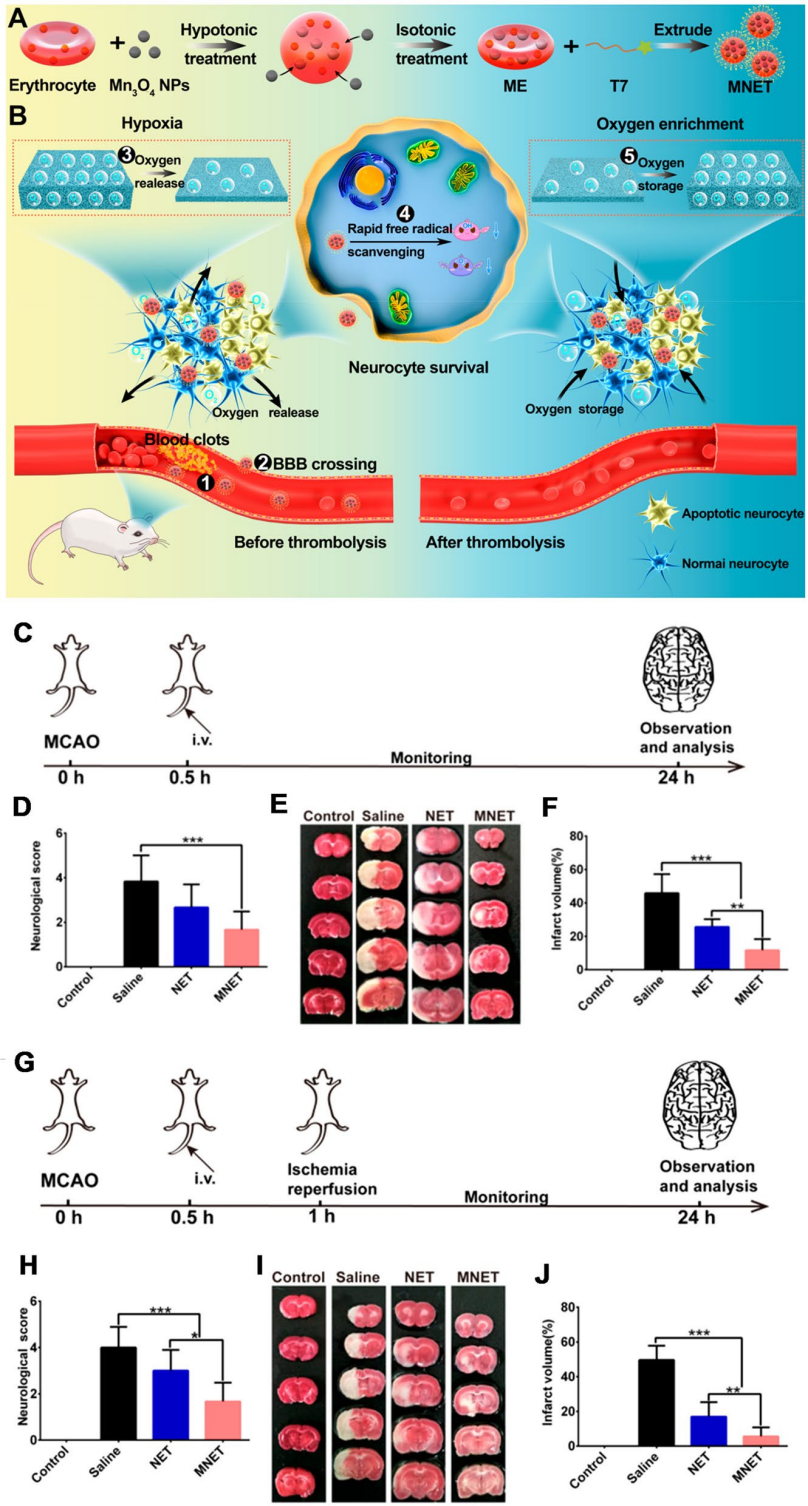
Schematic design and in vivo therapeutic effect of MNET before (C-F) or after (G-I) thrombolysis. **A** Preparation of MNET; **B** Principle scheme of MNET salvaging within an acute IS through the integration of free radical scavenging and natural oxygen sponge influence. **C** Timeline and design of animal experiments, NET and MNET were injected after 0.5 h post-MCAO surgery. **D** Neurological tests of MCAO rats received different treatments (n = 6). **E** Representative photographs of TTC-stained coronal brain slides and **F** quantitative analysis of infarct volume (n = 6). White areas in the ipsilateral hemisphere represent the infarcted regions. **G** Timeline and design of animal experiments, NET and MNET were injected after 0.5 h post-MCAO surgery. **H** Neurological tests of MCAO rats received different treatments (n = 6). **I** Representative photographs of TTC-stained coronal brain slides and **J** quantitative analysis of infarct volume (n = 6); White areas in the ipsilateral hemisphere represent the infarcted regions. Reproduced with permission from [181]. Copyright 2020, American Chemical Society

Erythrocytes, refer to the largest group of blood cells with significantly greater longer life-span as compared with other blood cells. Erythrocytes and erythrocyte membranes are quite easy to obtain, presenting novel insights into drug delivery for advanced targeting ability and biosafety as compared with artificial nanocarriers. Our group developed a bioengineered ROS-responsive nanocarrier for stroke-specific delivery against ischemic brain injury [180]. This biomimetic nanocarrier comprised a ROS-responsive boronic ester conjugated dextran polymer core with the modification of red blood cells (RBC) membrane shell under SHp insertion, thereby regulating the releasing of NR2B9C induced from high intracellular ROS within ischemic neurons. Moreover, Shi et al. presented an engineered nanosponge (Mn_3_O_4_@nanoerythrocyte-T7, MNET) capable of remodeling stroke microenvironment based on free radical scavenging as well as self-adapted oxygen control (Fig. 10). The bioengineered nanocarrier exhibited a prolonged circulation period in blood as impacted by the stealth influence exerted by the erythrocyte and preferential increase within the infarct site in two stages of an ischemic stroke: (i) before thrombolysis, rescue neurocyte *via* rapid free radical scavenging and timely oxygen supply; (ii) after thrombolysis, suppress oxygen-boost via oxygen storage [181], holding an attractive potential for IS treatment.

#### Platelets

**Fig. 11:**
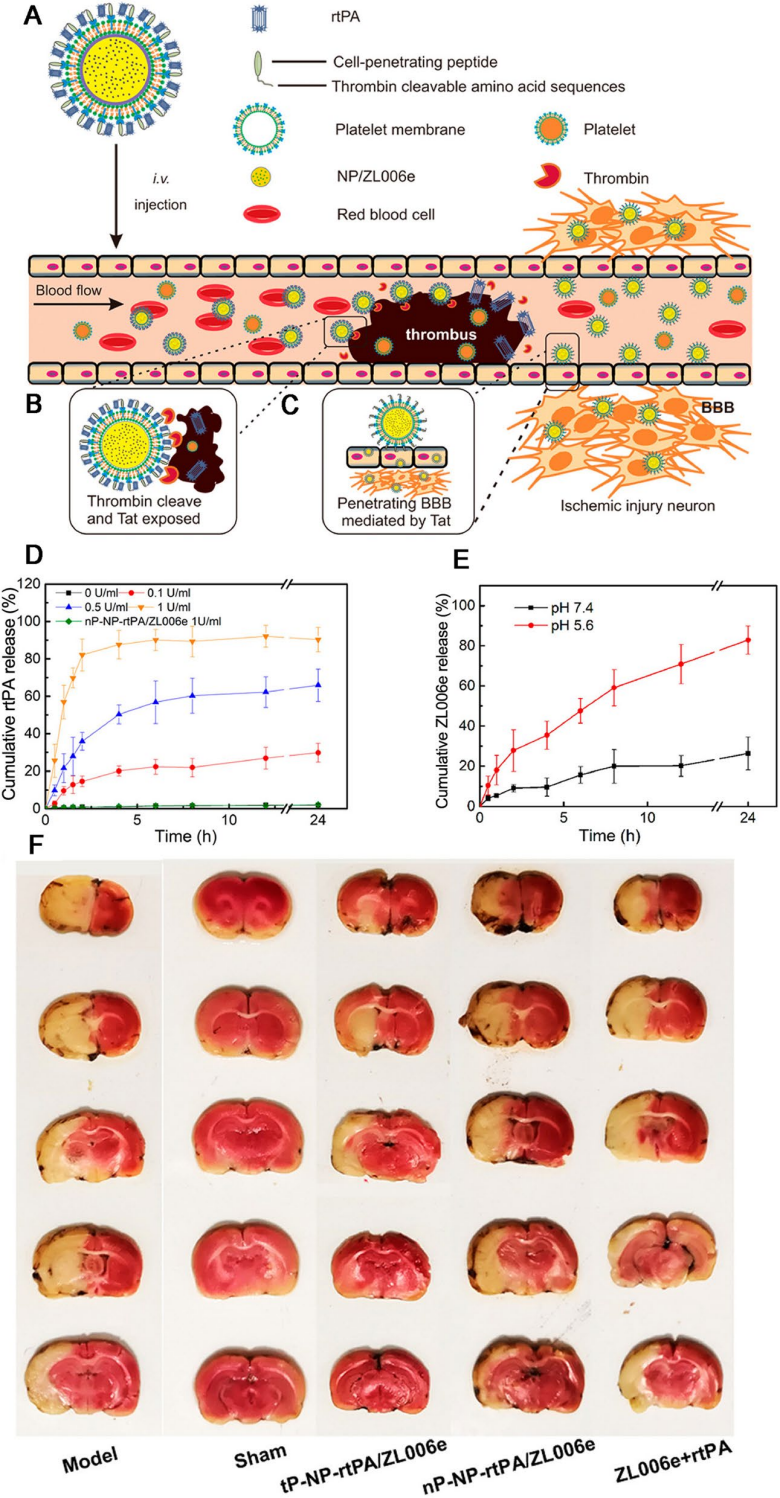
Schematic design and characterization of tP-NP-rtPA/ZL006e. **A** Main components of the tP-NP-rtPA/ZL006e. **B** After intravenous injection, tP-NP-rtPA/ZL006e was targeted to the thrombus for thrombin-triggered release of rtPA. **C** Nanocarrier transport into the brain via Tat-mediated transcytosis. **D** Cumulative release of rtPA from tP-NP-rtPA/ZL006e with different thrombin concentrations and nP-NP-rtPA/ZL006e in 1 U/mL thrombin. The reactions were performed in NaHCO_3_ (50 × 10^− 3^ M, pH 8.0) at 37 °C, with constant shaking. Error bars indicate standard deviation (n = 3). **E** Cumulative release of ZL006e from tP-NP-rtPA/ZL006e in phosphate-buffered saline with different pH levels at 37 °C, with constant shaking. Error bars indicate standard deviation (n = 3). **F** Representative TTC-stained brain sections of the MCAO model group, sham-operated group, tP-NP-rtPA/ZL006e group, nP-NP-rtPA/ZL006e group, and free ZL006e + rtPA group. The nonischemic area is observed as red, and the infarct area is shown in white. Reproduced with permission from [185]. Copyright 2019, American Chemical Society

Platelet, generated from megakaryocyte, refers to discoid, anucleate, small blood cells that are critical to hemostasis. Based on the platelets function in thrombus targeting and immune escape, platelets and platelets-like biomimetic nanocarriers play a role in stroke drug delivery to sites of vascular plaque [182]. In terms of small molecular drug, they are able to be ingested into platelets or combined to surface of platelets under the drug suspension incubation with the temperature of 37 °C [183]. This study loaded a biomimetic nanovesicles with L-arginine, a nitric oxide (NO) donor, and γ-Fe_2_O_3_ magnetic nanoparticles (PAMNs) for achieving curative purposes, taking into account the relevant role of natural platelets and thrombosis in targeting damaged vascular adhesion [184]. Our group later designed a thrombin-responsive platelet biomimetic nanoplatform (tP-NP-rtPA/ZL006e) for sequentially site-specific delivery of rtPA and ZL006e for IS treatment (Fig. 11). We reported that the nanoplatform could combine BBB targeting of ZL006e with thrombus-specific affinity of rtPA and have stimuli-induced releasing behavior, which was also demonstrated to significantly enhance the anti-ischemic stroke efficacy in MCAO rat model [185]. To solve the limited results of current thrombolysis approaches, Xu et al. developed an engineered nanoplatelets for targeting delivery of rt-PA to local site of thrombus. In pulmonary embolism and mesenteric arterial thrombosis model mice, the platelet membrane‐camouflaged polymeric nanocarriers (nanoplatelets) could rapidly increase at the thrombotic site, thus eliciting a noticeably promoted thrombolysis activity in comparison with free rt‐PA [186].

#### Leukocytes

Leukocytes, designed to accumulate in large numbers in areas of stroke injury and inflammation, can be significantly purified to carrier drugs. Inconsistent with erythrocyte and platelet, the distinguished property of neutrophils is chemotaxis, which endows them with the capability of migrating to an infection or inflammation site within minutes. According to several results, inflammation-related monocytes can be adopted to transport drugs to injury sites, whereas the blood monocyte number (nearly 5% of leukocytes) is insufficient for delivery of drug throughout the entire pathogenesis process of cerebral ischemia [187]. Compared to monocytes, neutrophils make up about 50% of white blood cells, making them the most abundant leukocytes in human body as well as a critical part of innate immunity. By hitchhiking on neutrophils to target the cerebral ischemia region, Wang et al. developed polymeric nanocarriers loading with catalase to cross BBB [188]. The mouse model of ischemia/reperfusion injury administrated by using the catalase-encapsulated nanocarriers indicated obvious enhancement of the curative outcome. According to the high curative efficacy and low side-effect of the neutrophil-targeted polymeric nanocarriers, the mentioned approach could show high prospect for eventual clinical transformation. Likewise, Dong et al. presented a drug delivery system consisting of neutrophil membrane-derived nanovesicles loaded with Resolvin D2 (RvD2) to enhance resolution of inflammation, thereby protecting against brain injury caused by IS [189]. Recently, our group exploited neutrophils as carriers for facilitating the BBB penetration of liposomes loaded with puerarin and elevating the puerarin concentration within the brain parenchyma [190]. The results indicated that puerarin-loaded liposomes could be released in response to inflammation-related conditions correlated to brain injury to enhance the neuro-protection effect of ischemic penumbra. This neutrophils-derived system has great potential and extensive value to provide an effective targeted drug delivery channel for poorly soluble drugs that have difficulty passing through the BBB. All the mentioned studies serve to establish the fact that the cell-derived biomimetic carriers provide effects and indicate the direction for ischemia therapy.

### Other functional nanocarriers

It is a key step for the drug therapy to temporarily control the drug release which can be of wide use in stroke therapy and satisfied the request of in vivo temporal and spatial selectivity of cargo release. Accordingly, the study of stimulus responsive carrier is almost inevitable for stroke treatment. A wide range of nanocarriers of natural and synthetic origin, organic and inorganic properties as well as with various shapes, sizes and surface modifications have been explored for drug delivery. In the context of cerebral stroke therapy, a number of unique responsive studies were applied in microenvironment of the brain. For instance, a H_2_O_2_-responsive engineered polymer nanoparticles formulated from copolyoxalate covering vanillyl alcohol (VA) (PVAX) was reported as a newly developed ischemia/reperfusion targeted nanotherapeutic agent [191]. The ROS-responsive charge-reversal poly[(2-acryloyl)ethyl (pboronic acid benzyl) diethylammonium bromide] (B-PDEA) polymer carrier was first developed by Jiang et al. to mediate efficient gene transfection in neural stem cells (NSCs), leading to efficient brain-derived neurotrophic factor (BDNF) expression and high curative efficacy in treating IS [192]. For the management of stroke, other reports about ROS responsive nanocarriers of stroke also emerged in an endless stream [193, 194]. Apart from the ROS microenvironment in brain, the stimuli-responsive nanocarriers based on pH or thrombin as a trigger switch have also been investigated for delivery of cerebral ischemia [185, 195]. Gao et al. explored a strategy of pH-triggered charge-conversion polymeric nanocarrier for DNA/protein delivery. They reported that the pH-regulated positively charged protein-coated polymeric micelles could effectively target the acidic environment and diagnostic imaging [196]. Stromal cell derived factor-1α (SDF-1α)-loaded pH-sensitive polymeric micelles have also been proved to be pH-triggered targeting agents that modify the microenvironment to enhance innate neural recovery processes [197]. Besides the ROS or pH, thrombin has also been explored to regulate the release of curative agents for stroke. For instance, Guo et al. reported a simple method of synthesizing ligand-conjugated, thrombin-responsive polymeric micells for drug targeted delivery to the ischemic brain [198]. This nanosystem was composed of block copolymers consisting of PEG, poly(ε-caprolactone) (PCL), and enzyme-cleavable peptides, noticeably enhancing the efficacy of glyburide for stroke treatment.

Some other nanomaterials with new features or multi-functions for IS treatment are emerging. For instance, Wang et al. have developed a near-infrared light (NIR)-driven nano-photosynthesis biosystem for rescue neurons in ischemic brain areas, achieving the new combined features of cell-biological, biochemical, and animal-level behavioral data [199]. Furthermore, the emergence of nanostructures with dual capabilities of therapy and diagnosis has given rise to a new field. The tPA-functionalized superparamagnetic ironoxide nanoparticles (SPIONs) were constructed as in vitro fibrin mimicking system. This biocompatible magnetite nanocarrier was designed to carry tPA for targeted thrombolysis under an external magnetic field, indicating the potential of future therapeutic applications in IS [200]. Similarly, a new strategy by incorporating tPA into magnetic microrods was attempted for simultaneous visualization by MRI and clot dissolution [201].

**Table 3:** Typical nanocarriers for treatment of IS

Nanocarriers	Categories of materials	Drugs or agents	Targeting ligands	Results	Refs.
Polymeric nanoparticles	Glutathione-coated PLGA-b-PEG	Thyroid hormones (T3)	Glutathione	Protect brain against ischemic injury	[89]
	Cationic lipid assisted PEG-PLA	C3-siRNA	_	Inhibit microglial neurotoxicity	[90]
	Chitosan	bFGF and z-DEVD-FMK	Transferrin	Reduce infarct volume in brain	[92]
	Tween80 coated chitosan-NIPAAM	Riluzole	_	Protect brain against ischemic injury	[93]
	_	Melanin	_	ROS scavenging and anti -inflammation-related	[96]
Polymeric micelles	Copolymer PEG-b-(PELG-g-PLL)	TNF-α		Attenuate the oxidative stress injury, the inflammation-related activity and the apoptosis level in I/R-induced cerebral injury	[104]
	Agonistic micelles	Edaravone	_	Regulate the BBB permeability and deliver neuroprotectants	[105]
Dendrimers	Arginine ester of PAMAM	siRNA	_	Perform siRNA-mediation gene knockdown in brain	[112]
	cationic PAMAM	_	_	Protect the injured brain from stroke by reaching the ischemic neurons	[110]
Nano-hydrogels	Alginate	VEGF	_	Induce significant functional and structural protection from ischemic injury	[119]
Liposomes	Phosphatidylethanolamine(PE), cholesterol, dicetylphosphate	CDP-Choline	_	Prevent age related global moderate cerebral ischemia reperfusion	[127]
	PEG-coated inositol hexaphosphate	Hemoglobin	_	Reduce ischemia reperfusion injury	[128]
	DPPC and cholesterol	Urokinase	Cyclic RGD	Improve the thrombolytic efficacy	[129]
	DPPC, cholesterol, PEG_2000_-PE	Minocycline	_	Reduce TNF-α induced MMP-9 release	[132]
	DSPC, DPPC, cholesterol, DSPE-PEG_2000_	FK506 (Tacrolimus)	_	Reduce cerebral cell apoptosis and ameliorate motor function deficits	[133]
	DPPC, Egg-PC, DPPG, cholesterol, PEG_2000_-PE	Xenon (Xe)	_	Reduce infarct size in brain	[135]
	Bean lecithin, cholesterol, DSPE-PEG_2000_	ZL006	Stroke homing peptide (SHp) and T7 peptide	Block nNOS-PSD-95 association and reduce infarct size in brain	[140]
Solid lipid nanoparticles	Monostearin, medium-chain triglyceride, polyethylene glycol monostearate	3-n-Butylphthalide	Fas ligand antibody	Improve brain injury and neurological function	[145]
	tripalmitin, Gelucires, vitamin E, phospholipids, and poloxamer 188	Baicalein		improve baicalein's stability and the ability of baicalein to penetrate the brain	[146]
	PEGylated cationic solid lipid nanoparticles	Baicalein	OX26	elevate biological availability of baicalin in cerebral spinal fluid of mice under the cerebral ischemia–reperfusion injury	[147]
Inorganic nanocarriers	Silica-coated superparamagnetic iron oxide	Endothelial progenitor cells (EPCs)	_	Improve neurobehavioral outcomes and reduce brain atrophic volume	[151]
	_	Amino-modificated carbon nano-scale tubes (CNTs)	_	Decrease cell apoptosis in the brain area	[162]
	Silica-coated Au or lipophilic polyaspartic acid-based polymer	Vitamin C	_	ROS scavenging	[163]
	_	Platinum	_	ROS scavenging	[164]
	_	Cerium oxide	_	ROS scavenging	[166]
Cell-derived biomimetic nanocarriers	Exosomes purified from BM-MSCs	microRNAs (miRNAs)	RVG-Lamp2b	Target delivery of gene drugs to the brain for ischemic brain	[174]
	Exosomes purified from BM-MSCs	Curcumin	c(RGDyK) peptide	Target delivery of curcumins to the brain for ischemic brain	[175]
	Mesenchymal stem cells (MSCs)	MiR-133b	Palmitic acid-peptide	Target delivery of miR-133b to increase the expression level in an ischemic lesion and further improve curative effects	[176]
	Dextran polymer core modified with RBC membrane shell	NR2B9C	Stroke homing peptide (SHp)	Reduce ischemia reperfusion injury	[180]
	Mn_3_O_4_@nanoerythrocyte	_	T7	Remodel the stroke microenvironment through self-adapted oxygen regulating and free radical scavenging	[181]
	γ-Fe2O3 magnetic nanoparticles inspired by natural platelets	L-arginine	_	Target adhesion to the injured brain blood vessel during formation of thrombus	[184]
	Acetal Modified Dextran modified with platelet membrane shell	ZL006e and tPA	Cell-penetrating peptide	Enhance the treatment of thrombolytics and neuroprotectant for IS	[185]
	Neutrophil-mediation cross-linked dendrigraft poly-L-lysine (DGL) nanoparticles	Catalase	PGP ligands	Protect the catalase enzymatic activity from degradation and well transport to receiver cells	[188]
	Neutrophil membrane-derived nanovesicles	Resolvin D2 (RvD2)	_	Enhance resolution of inflammation, thus protecting brain injury during IS	[189]
	Neutrophils-derived liposomes system	Puerarin	-	Enhance the neuro-protection effect at the ischemic penumbra	[190]
Other functional nanocarriers	Copolyoxalate (H_2_O_2_-responsive)	Vanillyl alcohol(VA)	_	Exert anti-inflammation-related and anti-apoptotic activities	[191]
	B-PDEA (ROS-responsive)	Neural stem cells (NSCs)	_	Lead to efficient expression of brain derived neurotrophic factors	[192]
	PLA-coated mesoporous silica (ROS-responsive)	Resveratrol	Low-density lipoprotein receptor (LDLR)	Enhance the transcytosis across the blood–brain barrier	[193]
	Methoxy poly(ethylene glycol)-poly(L-ethionine) diblock copolymers (H_2_O_2_-responsive)	Rhodamine-6G	_	Serve as promising platforms for sustained drug delivery for diseases with local oxidative stress	[194]
	Methoxy poly(ethylene glycol)-poly(β-amino ester) (PEG-PAE) with piperidine and imidazole rings (pH-responsive)	Human serum albumin (HSA)	_	Target the acidic environment in brain	[196]
	Poly (urethane amino sulfamethazine) (PUASM) (pH-responsive)	Stromal cell derived from factor-1α	_	Modify the microenvironment to increase innate neurorestorative processes	[197]

## Summary and conclusion

To conclude, IS is a mental medical emergency which happens due to obstruction of blood supply (blockage of vasculature) to specific regions of the brain. The pathophysiology mechanism of IS involves a complex dynamic process, primarily including glutamate excitotoxicity, disruption of Ca^2+^ homeostasis, oxidative stress attributed to ROS generation and cerebral inflammation, which remains a great challenge for clinicians because of the critical concerns involved in its curative window, presence of BBB as well as long-term effects of impaired brain function. The lack of approaches for efficient drug delivery to the brain is one of the main difficulties to develop effective treatment for IS. Besides, owing to the non-specificity, slow onset of action, narrow therapeutic efficacy, and poor patient compliance, clinical value of these conventional medications (i.e., thrombolytic and antiplatelet agents, neuroprotective agents) is limited.

Nowadays, application of nanocarriers (including polymer-based nanocarriers, lipid-based nanocarriers, inorganic nanocarriers, Cell-derived biomimetic nanocarriers) for delivering curative agents is gaining more and more attention as a possible alternative to traditional therapy due to the following points: (i) reduce recognition and clearance by reticuloendothelial system (RES) and prolong the blood circulation time in vivo; (ii) improve the blood stability and enhance the BBB penetration and lesion site targeting; (iii) reduce the off-target toxicity of therapeutic agents; (iv) co-deliver therapeutic agents with different targets for synergistic therapy; (v) achieve the stimulus-reaction (response to ROS or pH, inflammatory markers, etc.). In addition, this nanotechnology also allows for better high resolution visualization of infarct sites, greatly promoting the diagnosis and treatment of IS.

Despite the rapid development and potential clinical prospects of nano-delivery system, several difficulties of nanocarriers need to be emphasized in the near future and their clinical transformation is greatly challenged. Firstly, in view of the complex pathological mechanisms of IS, the nanocarriers usually only target one or two pathological mechanisms and fail to provide comprehensive protection for the CNS. Co-delivering different promising drugs into a single nano-delivery system indeed provides a possibility of multifaceted protection, but it inevitable increases the complexity of the nanocarriers and hampers their clinical translation. Secondly, there are many studies on the anti-stroke of nanocarriers, but most of them just focus on the consideration of drug delivery effectiveness. The mechanism of cerebral metabolism and elimination of nanocarriers as well as the long-term safety for clinical application, are still unclear and the correlated neurotoxicity is yet a matter of concern. The safety data and toxicity profiles of nanocarriers after internalization and retention in brain tissues are not attracted enough attention. Due to the toxicity of nanocarriers is a critical factor affecting its clinical transformation, toxicity investigation of nanocarriers should become an important direction for scientists in this field in the further. Thirdly, although BBB penetration and ischemic penumbra focusing effect of nanocarriers can be enhanced by brain-targeted modification or biomimetic approaches, the BBB still remains a major challenge, and a large proportion of nanocarriers still fail to reach the ischemic area. In particular, the pathogenesis of model animals (usually MCAO model) is not exactly the same as that of clinical patients, leading to the scarcity of clinical evaluation data of existing nanomedicines in human body, which also explains why is tough to realize clinical translation of nano-drug delivery carriers.

Looking forward, more in-depth researches are urgently demanded and further studies should be pursued to reveal the actual efficiency of drug delivery for IS and offer more insight on how to achieve the translation of the drug-loaded nanosystems successfully. Despite the above concerns, it is self-evident that nanotechnology is the way forward in diagnosing, treating and managing IS.

## Abbreviations

IS: Ischemic stroke
MCAO: Middle cerebral artery occlusion
AHA: American Heart Association
tPA: Tissue-type plasminogen activator
BBB: Blood–brain barrier
CBB: Cerebrospinal fluid barrier
CNS: Central nervous system
ROS: Reactive oxygen species
RONS: Reactive oxygen species and nitrogen species
NO: Nitric oxide
NMDA: N-methyl-D-aspartic acid
AMPA: α-Amino-3-hydroxy-5-methyl-4-isoxazole-propionicacid
IAP: Inhibitors of apoptotic protein
PARP: Poly-(ADP ribose) polymerase
AIF: Apoptosis-inducing factor release
iNOS: Inducible nitric oxide synthase
GABA: γ-Aminobutyric acid
EDTA: Ethylenedimainetetraacetic acid
BCECs: Brain capillary endothelial cells
BMECs: Brain microvasular endothelial cells
NSCs: Neural stem cells
BMDSC: Bone marrow derived stem cells
MCS: Mesenchymal stem cells
iPSC: Induced pluripotent stem cells
RBC: Red blood cells
NPs: Nanoparticles
PNP: Polymeric nanoparticles
CNTs: Carbon nano-scale tubes
nPts: Platinum nanoparticles
OGD/R: Oxygen–glucose deprivation/reperfusion
NIPAAM: N-isopropylacrylamide
MeNPs: Melanin nanoparticle
MNPs: Magnetic nanoparticles
PAMAM: Polyamido amine
VEGF: Vascular endothelial growth factor
BDNF: Brain-derived neurotrophic factor
RVG: Rabies virus glycoprotein
Lamp2b: Lysosome-correlated membrane glycoprotein 2b
SDF-1α: Stromal cell derived factor-1α
PCL: Poly(ε-caprolactone)
RES: Reticuloendothelial system
PET: Positron emission spectroscopy
CT: Computed tomography
DSA: Digital subtraction angiography
SPECT: Single photon-emission computed tomography
CT-A: CT angiography
CT-P: CT perfusion
SPIO: Superparamagnetic iron oxide
SPIONs: Superparamagnetic ironoxide nanoparticles
TEM: Transmission electron microscope
MRI: Magnetic resonance imaging
